# Potent phytochemicals against COVID-19 infection from phyto-materials used as antivirals in complementary medicines: a review

**DOI:** 10.1186/s43094-021-00259-7

**Published:** 2021-06-02

**Authors:** C. S. Sharanya, A. Sabu, M. Haridas

**Affiliations:** Inter University Centre for Bioscience and Department of Biotechnology & Microbiology, Dr. Janaki Ammal Campus, Kannur University, Thalassery, 670661 India

**Keywords:** COVID 19, SARS CoV-2, Spike protein, Main protease, Polyphenols, Traditional Chinese Medicine, Indian system of medicine, Ayurveda and Siddha

## Abstract

**Background:**

Following the outbreak of the COVID-19 pandemic, there was a surge of research activity to find methods/drugs to treat it. There has been drug-repurposing research focusing on traditional medicines. Concomitantly, many researchers tried to find in silico evidence for traditional medicines. There is a great increase in article publication to commensurate the new-found research interests. This situation inspired the authors to have a comprehensive understanding of the multitude of publications related to the COVID-19 pandemic with a wish to get promising drug leads.

**Main body:**

This review article has been conceived and made as a hybrid of the review of the selected papers advertised recently and produced in the interest of the COVID-19 situation, and in silico work done by the authors. The outcome of the present review underscores a recommendation for thorough MDS analyses of the promising drug leads. The inclusion of in silico work as an addition to the review was motivated by a recently published article of Toelzer and colleagues. The in silico investigation of free fatty acids is novel to the field and it buttresses the further MDS analysis of drug leads for managing the COVID-19 pandemic.

**Conclusion:**

The review performed threw light on the need for MDS analyses to be considered together with the application of other in silico methods of prediction of pharmacologic properties directing towards the sites of drug-receptor regulation. Also, the present analysis would help formulate new recipes for complementary medicines.

## Background

This article has been conceived and made in a hybrid model; a hybrid comprising of a review of the selected papers advertised recently and produced in the interest of the COVID-19 situation, and a small amount of in silico unpublished work done by the authors regarding the pandemic too. The authors’ work was motivated to be included in this article by a recently published article by Toelzer and colleagues [1]. A review of the protein structures significant in COVID-19 management by medicines has been included as part of this article.

The in silico studies considered in this review are from groups who used different programmes for docking and simulation works. Consequently, the results obtained from such studies using different program packages could not be compared as numerical equivalents of obtained/calculated valued. So, their comparison would amount to qualitative assessment only. Hence, they are not considered for deriving any direct recommendation for suggesting any in vivo assessment of anti-SARS-CoV-2 virus application as a result of this review. Since few in vitro studies did appear in the referenced research articles, they are deficient for finding the best ones out of proposed drug leads having potential for carrying forward into in vivo experiments. However, there is a right take-home message that the traditional medical methods of managing influenza expressing symptoms quite similar to SARS-CoV-2 also need to be considered treatment strategies and/or for drug development.

## Main text

Since late 2019, a respiratory syndrome cluster has been observed in the Wuhan province of China caused by a novel beta coronavirus and the disease spread all over the world in a few months. This world disease was spread through the deadly virus, and it was found that the disease was a modified form of the severe acute respiratory syndrome (SARS), which was overruled in late 2010. The enormous features of coronaviruses are that they cause mild to severe infections in humans as we could see the case of SARS and Middle East Respiratory Syndrome (MERS). This contagious virus is coming under the family Coronaviridae (subfamily Coronavirinae) and the members of which infect a broad range of hosts, producing symptoms and diseases ranging from the common cold to severe and often fatal illnesses, such as SARS, MERS, and the present coronavirus disease-19 (COVID-19) [2]. WHO announced at the beginning of 2020 that this infection is a pandemic. WHO officially designated the causative agent as SARS-CoV-2 and the disease was named COVID-19. The first observation of this disease came from workers in the Chinese Huanan seafood and wholesale live animal market, and it was believed that this disease was transmitted from animals. Coronavirinae, the subfamily of the novel coronavirus, is classified into four genera alpha, beta, gamma, and delta. The novel coronavirus belongs to the beta coronavirus group, and the other members include SARS HCoV (human coronavirus), HCoV-OC43, and MERS-CoV, whereas the alpha corona group includes HCoV-NL63 and human coronavirus (HCoV)-229E and the virus isolated from whales, birds, and pigs are included in the groups of gamma and delta coronaviruses respectively [3].

### COVID-19 outbreak: a brief history and pathology

**Fig. 1:**
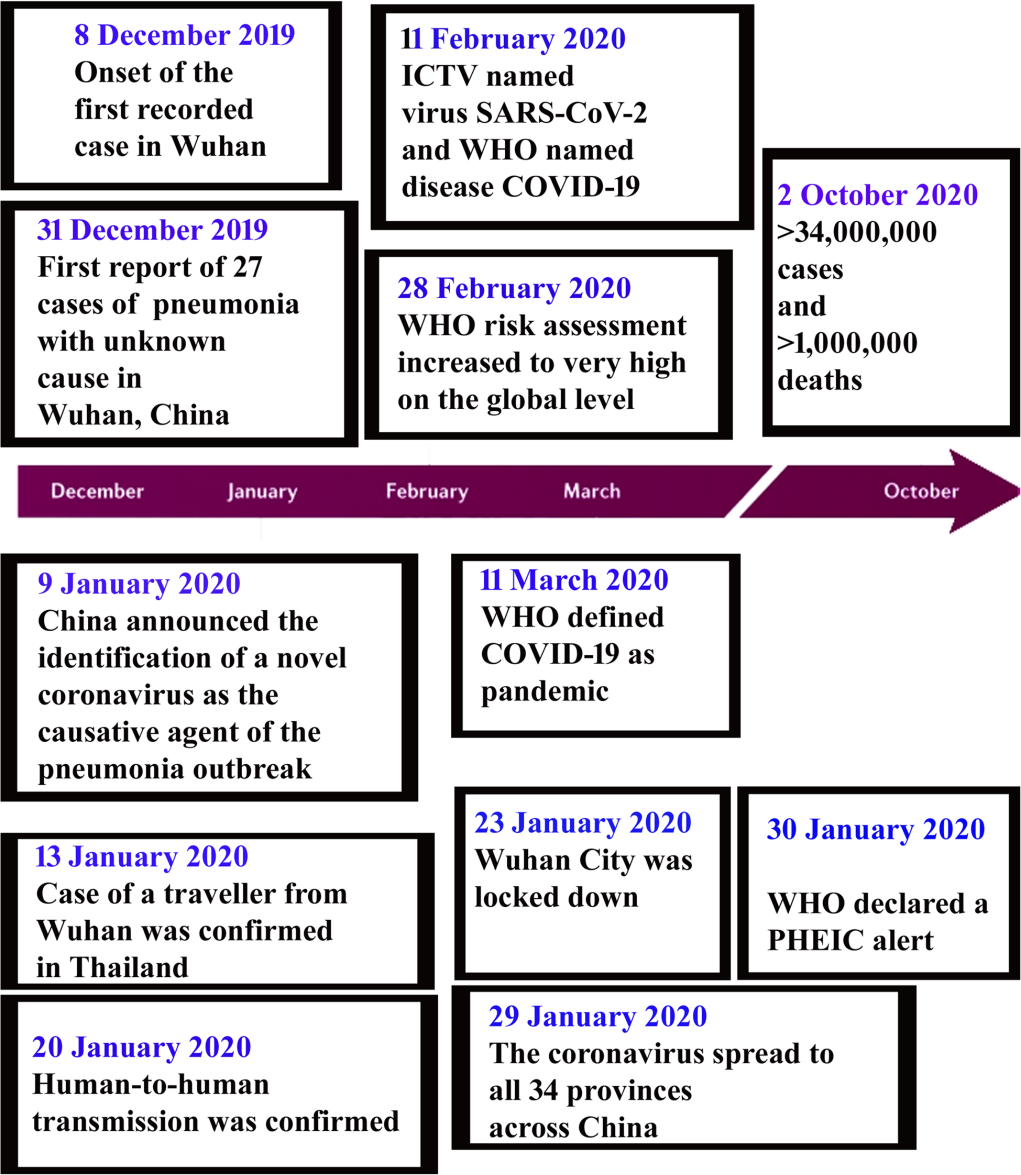
The brief history of the outbreak

The first outbreak was reported in early December 2019 with many clusters. The first 27 patients were admitted with unknown viral pneumonia. The epidemiological study showed that they were linked to the seafood, poultry, and wild animal wholesale market in Huanan, China [4]. The Chinese government reported the disease to WHO on December 31st as viral pneumonia with an unknown cause. On January 2nd, 41 patients were admitted due to severe symptoms, including ARDS, fever, and myalgia [5]. It was advised that those who showed symptoms of ARDS should be immediately hospitalized. They may need oxygen therapy and an ICU facility. Late hospitalizing, even within 2 days of ARDS, might become fatal. Six patients out of 41 admitted in the hospital died during the early stage of their hospitalization. Later on, the virus-positive cases increased rapidly, and the death cases swelled [5] (Fig. 1).

**Fig. 2:**
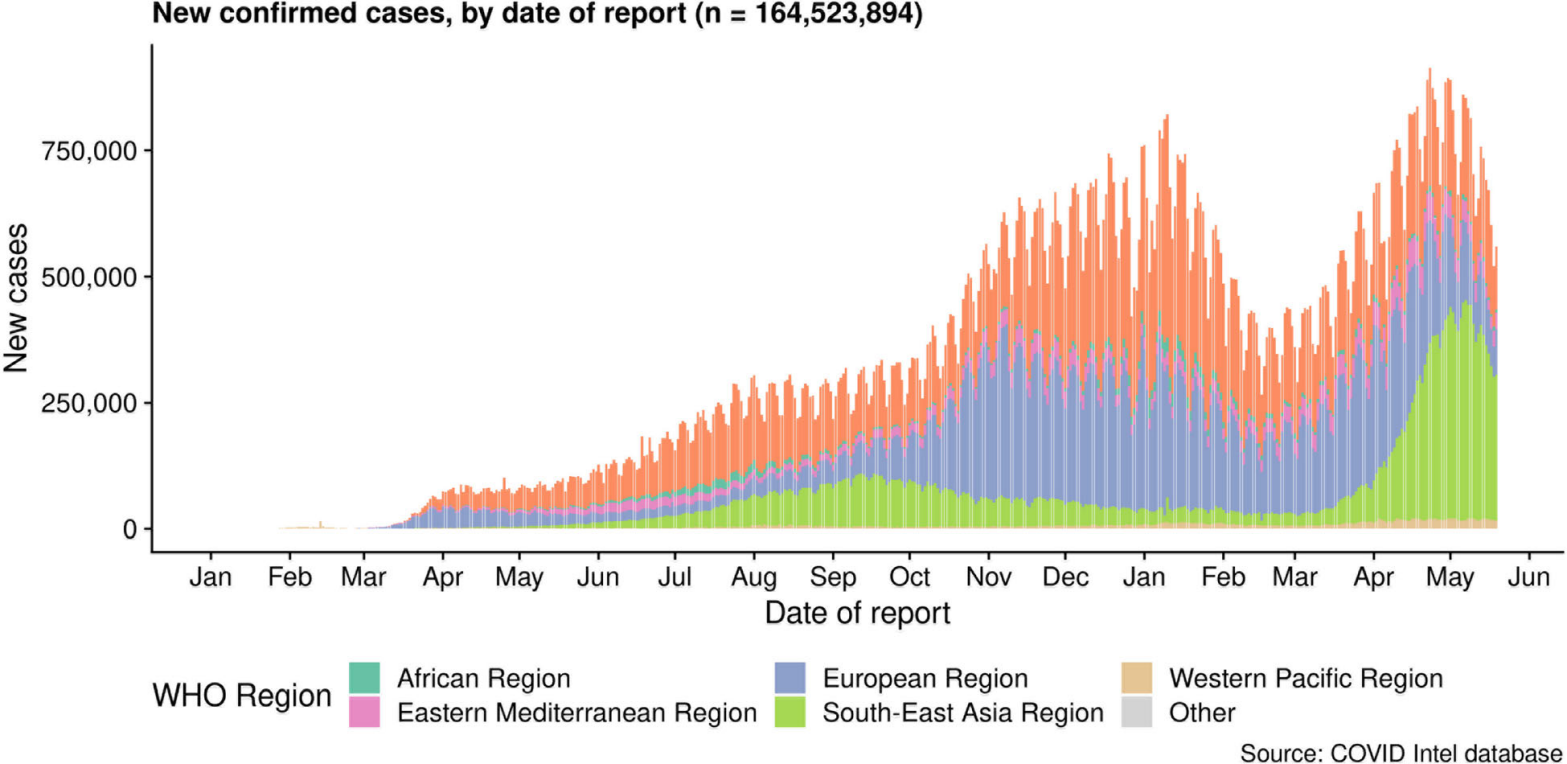
The reported covid-19 cases all over the world (WHO Case study) [7]

The disease was transmitted from human to human within a short period, and it spread over to other provinces of China too. Not only in China had it spread all over the world through travellers in and out of China to other countries (WHO). The only and quick way to prevent transmission of the disease is social distancing. Thus, the Chinese government declared a complete lockdown of the cities and restrictions on travelling, public gathering, functions, etc. However, within that period, disease spread to other countries and the second country with great ruin occurred in Italy [6]. Italy became the first European country which was greatly affected earlier with COVID-19, and the first case report came on January 30th. In China, the disease transmission was very high during February and applying strict public measures, and other travel/transportation blocking, led to a decrease in the active cases. Instead of this decrease, internationally, the spread reached its maximum. WHO reported that, from 2019 December 30th through 2021 April 19th, over 140 million COVID-19 cases and 3 million deaths had occurred globally (Figs. 1 and 2). Nearly half of these cases (48%) and deaths (55%) were reported with the USA, Brazil, and Argentina and new increased cases from India are reporting nowadays (Figs. 1 and 2) [7].

The patients with SARS and MERS have extensive lung damage with pulmonary inflammation and an increased concentration of pro-inflammatory cytokines like IP10, IL12, IL1B, IL6, IFNγ, IL15, IL17, MCP1, and TNFα in the serum [8–10]. Similarly, COVID-19 patients showed activated T-helper-1 (Th1) cell responses as a result of increased concentration of IL1B, IFNγ, IP10, and MCP1 [5]. Later on, patients admitted in the ICU with serious ill effects showed enhanced amounts of GCSF, IP10, MCP1, MIP1A, and TNFα than patients without ICU. It means that the cytokine storm might be responsible for the severity of the disease [11, 12]. Also, the haematology results of patients with mild severity of COVID-19 showed a decreased amount of lymphocyte count and reduction in natural killer cells, CD^4+^ T cells, and CD^8+^ T cells. In death cases, an extensive decline in the Th populations was observed. Moreover, the T cell subsets which produce pro-inflammatory molecules like IL-17 were also increased and resulted in disease severity. In addition to this, secondary haemophagocytic lymphohistiocytosis (HLH) might be associated with COVID-19, the disease related to cytokine storm [13]. The disease was progressed by the histiocytes that exhibit haemophagocytic activity or expansion of tissue macrophages. The cause of HLH is a defect in the cytolytic pathway, which is genetic and also due to other states like rheumatic diseases, malignancies, and infections [14].

### Structural characteristics of SARS-CoV-2

Studies on the metagenomic RNA sequencing of the virus revealed that this was a new beta coronavirus that has been found never in history, causing the disease, and its genome sequence is now publicly available [15, 16]. This novel coronavirus shares 79% structural similarity with SARS-CoV and 50% with MERS-CoV. The main structural features include open reading frames starting from 5′-3′: ORF1a/ORF1b (replicase), envelope, nucleocapsid, spike, and membrane. In between these structural genes, other proteins encoded for seven putative open reading frames are also embedded. Furthermore, 16 non-structural proteins (nsp 1–16) cleaved by the main protease (M^pro^, also referred to as 3CLpro) and the papain-like protease PLpro from large polypeptides encoded by replicase are responsible for viral replication [17]. The phylogenetic analysis by Jaimes et al. [18] showed that the SARS-CoV-2 S protein is closely related to the bat coronavirus (BatCoV-RaTG13) having 99% similarity, and this group is closest to SARS-CoV-2. Nevertheless, direct transmission from a bat is not yet proven [19, 20]. Hence, the intermediate reservoir for SARS-CoV-2 was not proven, and the study showed that the spike sequence in pangolin is grouped in a subclade branching from RaTG13 [21].

### Phyto-inhibitors of proteins relevant in SARS-CoV-2 infection

The main antigenic part of the SARS-CoV-2 is the S protein. Targeting the S protein may produce an excellent therapeutic lead in the drug discovery process. This antigenic part of SARS-CoV-2 induces the immune system of the host, and neutralizing antibodies could be used to protect against the infection. There are ongoing studies with these targets and vaccines are under the developing stage. Similar to the vaccine, other drug molecules are also tried for treating these viral diseases. Drug repurposing is another method where we may repurpose the already available drugs to SARS-CoV-2. Thus, we may reduce the time of experimenting with pharmacokinetic properties. Already available drugs passed through all the drug evaluation protocols could reduce the drug discovery time. For example, the major drug molecules like lopinavir [22–24], remdesivir [25–27], favipiravir [28–31], ritonavir [24, 32, 33], ribavirin [34, 35], interferon (IFN) [36, 37], nebulized α-interferon [30, 38], chloroquine [39–41], and hydroxychloroquine [42–45] are suitable to treat SARS-CoV-2 infection. Nevertheless, there are side effects and toxic effects while using these drugs and safe and effective drugs with few side effects should be developed. Such repurposing is an immediate imperative for tiding over the SARS-CoV-2 pandemic. However, for application in the long run, specific drugs for SARS-CoV-2 infection must be developed. The above suggests the use of herbal preparations and plant-based drug molecules may be developed based on evidence. They may be more beneficial and with reduced risk factors related to drug applications [46].

Drug repurposing may be used as a methodology where we can reduce the time lag for identifying new drugs. But, there are certain limitations in using these drugs alone or in combination. Most of the approved drugs showed certain side effects like nephrotoxicity, hematologic toxicity, cardiotoxicity, and hepatotoxicity. The promising antimalarial drugs chloroquine and hydroxychloroquine resulted in cardiomyopathy, neuromyopathy, and retinopathy. In the earlier phase of drug discovery against SARS CoV-2, CQ and HCQ are found promising. Later on, studies proved decreased in-hospital survival and increased frequency of ventricular arrhythmias in hospitalized patients. Hence, the trial with these drugs was suspended and WHO notified that HCQ has no beneficial effect and discontinued its use for COVID-19 infection with immediate effect [45, 47–49].

**Fig. 3:**
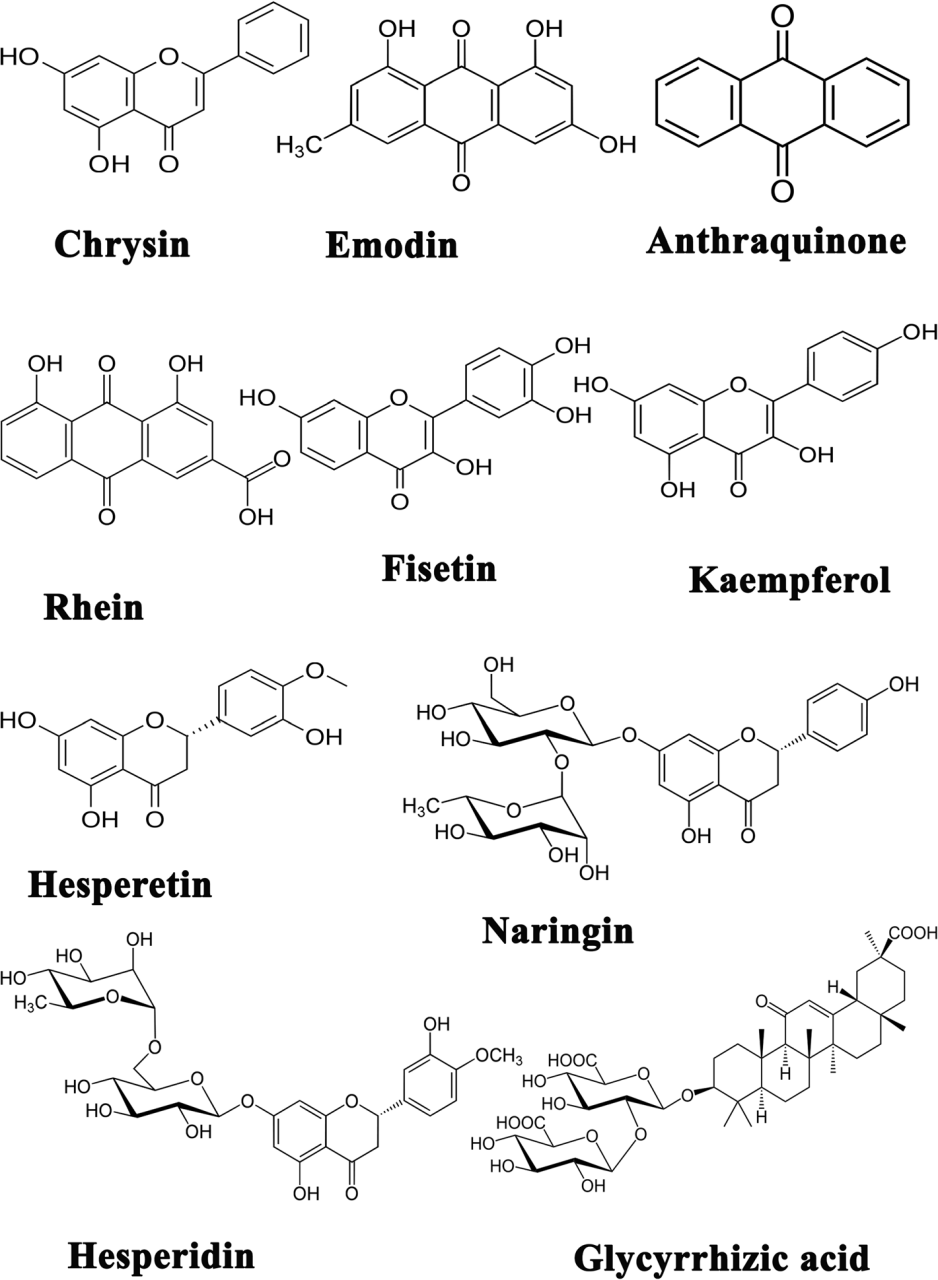
Antiviral flavonoids suggested as spike-ACE2 complex inhibitors

Phytochemicals with increased bioactivity and little toxicity may be the most efficient alternative way for treating most of the diseases [50]. Since ancient times, people have depended on the utilization of naturally available materials, particularly phytochemicals (herbal extracts as crude, or treated extracts, concoctions, or decoctions), minerals, and animal products, for treatments of various ailments and other health problems [51]. Hence, the importance of plant secondary metabolites has come into experts’ notice. The major phytocompounds are flavonoids, terpenoids, alkaloids, and phenolic and essential oils [52]. Here, the most striking model may be the use of quinine, an alkaloid from the bark of the cinchona tree, utilized to treat malaria. It was also used to treat different infectious diseases, like typhoid fever, pneumonia, and even conventional nasopharyngeal infections [53]. The antiviral nature of plant secondary metabolites is also described indirectly in ancient medical texts. There are numerous mechanisms which direct the antiviral activity of phytochemicals, like interfering with the viral replications by targeting DNA/RNA polymerase, jeopardizing viral assembly or post-translational modification, etc. established in the last century [51, 54]. The genome sequence similarity between SARS-CoV and SARS-CoV-2 could be used effectively and fruitfully in searching for better drug molecules, where those used to treat SARS could be used to treat SARS CoV-2. Hence, previously reported plant components against SARS can be explored to use/repurpose (Fig. 3).

The generally used phenolic subclasses for treating viruses are phenolic acids, flavonoids, stilbenes, coumarins, and tannins. The main targets found are influenza virus [55], Zika virus [56], chikungunya virus [57], rotavirus [58, 59], dengue virus [60], hepatitis virus [61], Japanese encephalitis virus [62], herpes virus [63], human papillomavirus [64], and human immunodeficiency virus (HIV) [64].

A recent report showed [65] that the flavonoids fisetin, quercetin, and kaempferol would bind to the spike protein and can be a suitable lead molecule against SARS-CoV-2. Their study is based on the data generated by in silico molecular docking studies. They could identify the most potent inhibitor from selected flavonoid and non-flavonoid compounds (kaempferol, curcumin, pterostilbene, hydroxychloroquine, fisetin, quercetin, isorhamnetin, genistein, luteolin, resveratrol, and apigenin). Docking studies were carried out with the cryo-EM structure of the spike protein available in the PDB, and its A chain was selected as the receptor for the study (PDB ID: 6VYB). In silico studies of antimalarial drug hydroxychloroquine also showed efficacy against SARS-CoV-2. The molecular docking and binding affinity results showed that fisetin, quercetin, and kaempferol have − 8.5 kcal/mol, − 8.5 kcal/mol, and − 7.4 kcal/mol binding affinity respectively than HCQ (− 5.6 kcal/mol). Hence, the docking analyses of these molecules were taken forward to molecular dynamics studies for analysing the stability at the target and conformational changes if any. The simulation study was carried out with hACE2–S protein complex bound with fisetin, quercetin, and kaempferol. The results showed that the phytocompounds bound to the hACE2-S complex with low binding free energy and suggested that they can interfere with the complex and inhibit the viral entry and further signal cascades.

Stilbene-based natural compounds are potent inhibitors of spike glycoprotein-ACE2 complex [66]. Stilbenoids are phytoalexins, which are synthesized during injuries, in response to fungal or bacterial toxins and UV radiations. These phenolic compounds have several biological functions, and one of the important stilbenes is resveratrol. The molecular docking studies showed that piceatannol has a better docking affinity as compared to the complex having resveratrol. However, simulation results deviated from the docking results in this case and proved resveratrol-protein as the most stable and suitable complex for further analysis. The conflicting interaction proceeding towards the resultant binding of the ligand to target, learnt from molecular dynamics simulation, is not an unnatural feature of particular biological reaction/interaction. However, the take-home message from the conflicting results of molecular docking and molecular dynamics simulation studies would be suggesting of more or less the same result, unless in suicide inhibition. Dithymoquinone in *Nigella sativa* significantly inhibited the spike glycoprotein–ACE2 interface with a binding affinity of − 8.6 kcal/mol than the positive control chloroquine, − 7.2 kcal/mol. *Nigella sativa* seeds are known for treating various ailments including inflammation, asthma, bronchitis, fever, pain, hypertension, and eczema [67]. Recent studies showed that glycyrrhizic acid is also a prominent lead molecule to treat COVID-19. Glycyrrhizic acid is a saponin having a potent anti-inflammatory effect and immune-modulatory activity which shows both cytoplasmic and membrane effects. Studies have shown that glycyrrhizic acid is a broad-spectrum anti-coronavirus candidate with low toxicity. The binding activity of glycyrrhizic acid at the interface of the spike protein RBD–ACE2 explained via the surface plasmon resonance and nano bit assay. The experimental protocol revealed that glycyrrhizic acid was an efficient inhibitor. Cytotoxicity studies had proven its non-toxic nature [68, 69]. The flavonoid molecules like hesperidin, emodin, anthraquinone, rhein, and chrysin had also been studied against the spike protein. The protein model of the spike RBD sequence was modelled and this protein used for binding studies with ACE2. The protein bound with ACE2 was used as the drug target for simulation studies. Hesperidin, emodin, and chrysin showed potential activity against the drug target described above [70].

**Fig. 4:**
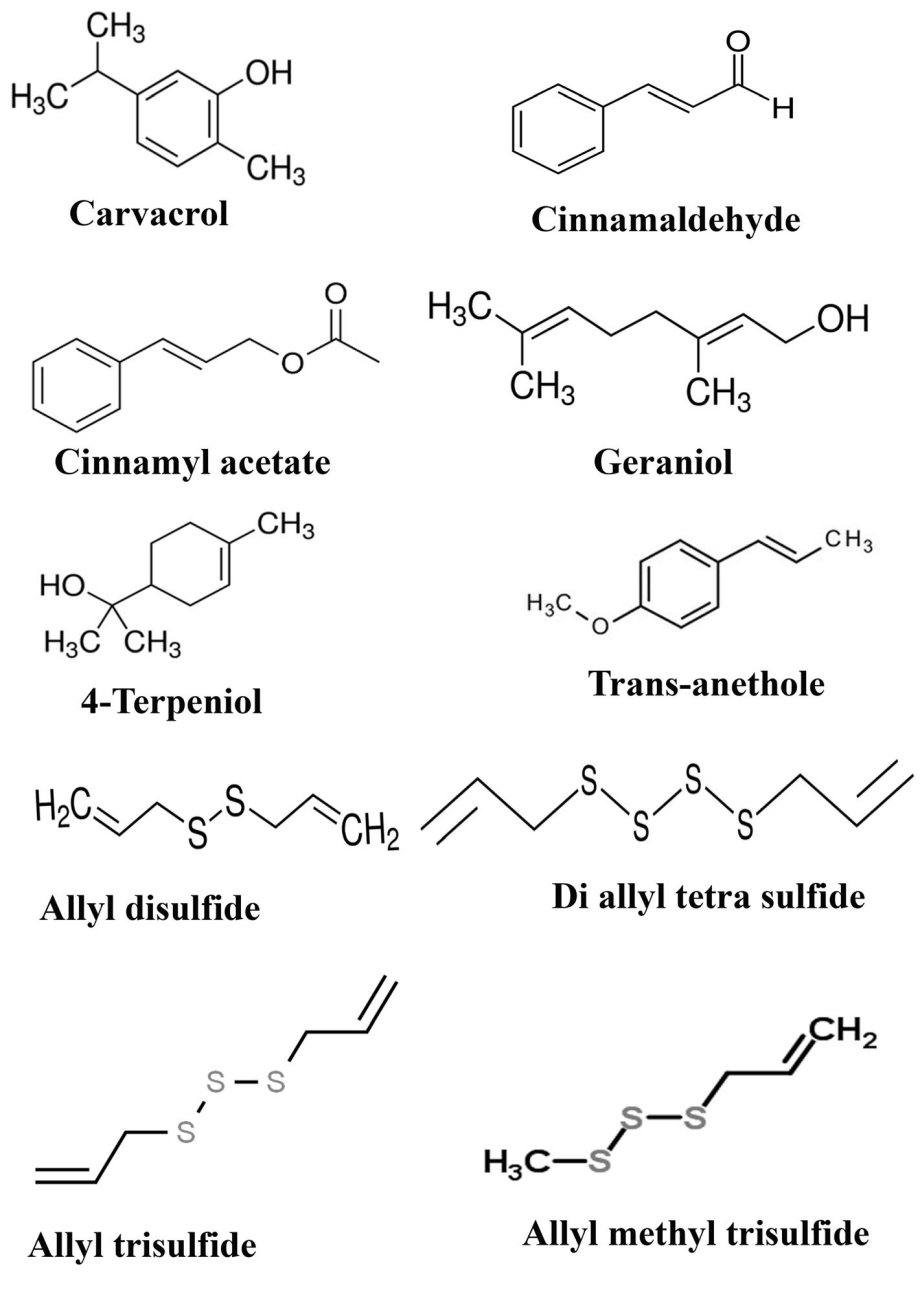
Essential oils suggested as spike protein inhibitors

Essential oils may form good candidates for drug discovery attempts due to their non-toxic nature and their simplicity to use (Fig. 4). Certain essential oil compounds from cinnamon, clove, thyme, star anise, basil, holy basil, eucalyptus, geranium, oregano, and ajwain were analysed against the spike protein. The receptor domain residues interacting with ACE2 were Tyr449, Tyr453, Asn487, Phe486, Tyr489, Gln493, Gly496, Gln498 Thr500, Gly502, and Tyr505. Carvacrol, cinnamaldehyde, cinnamyl acetate, geraniol, l-4-terpineol, and anethole displayed better binding affinity towards the target protein, and the protein–ligand complexes were stabilized by hydrogen bonds, hydrophobic interactions, etc. [67].

The essential oil component of garlic showed a potent inhibitory effect against the SARS-CoV-2 receptor ACE2. The major constituents of the essential oil of garlic are organosulfur compounds having many pharmacological activities. Studies have shown that these compounds have good interaction with ACE2. The GC-MS analysis of essential oil revealed the presence of 18 compounds like allyl disulphide (28.4%), allyl trisulphide (22.8%), allyl (E)-1-propenyl disulphide (8.2%), allyl methyl trisulphide (6.7%), and diallyl tetrasulphide (6.5%) [71]. These molecules were docked against ACE2 and their dynamics simulation studies carried out. The organosulphur compounds have shown strong binding interactions with the amino acids Pro565, Trp566, Ala396, Gln102, Gln101, Glu208, Gly205, Gln98, Asn210, Lys94, Lys562, Val209, and Ser563 and most of the compounds have shown the same results. It could be that the essential oil of garlic shall be a good material for treating COVID-19 [71].

**Fig. 5:**
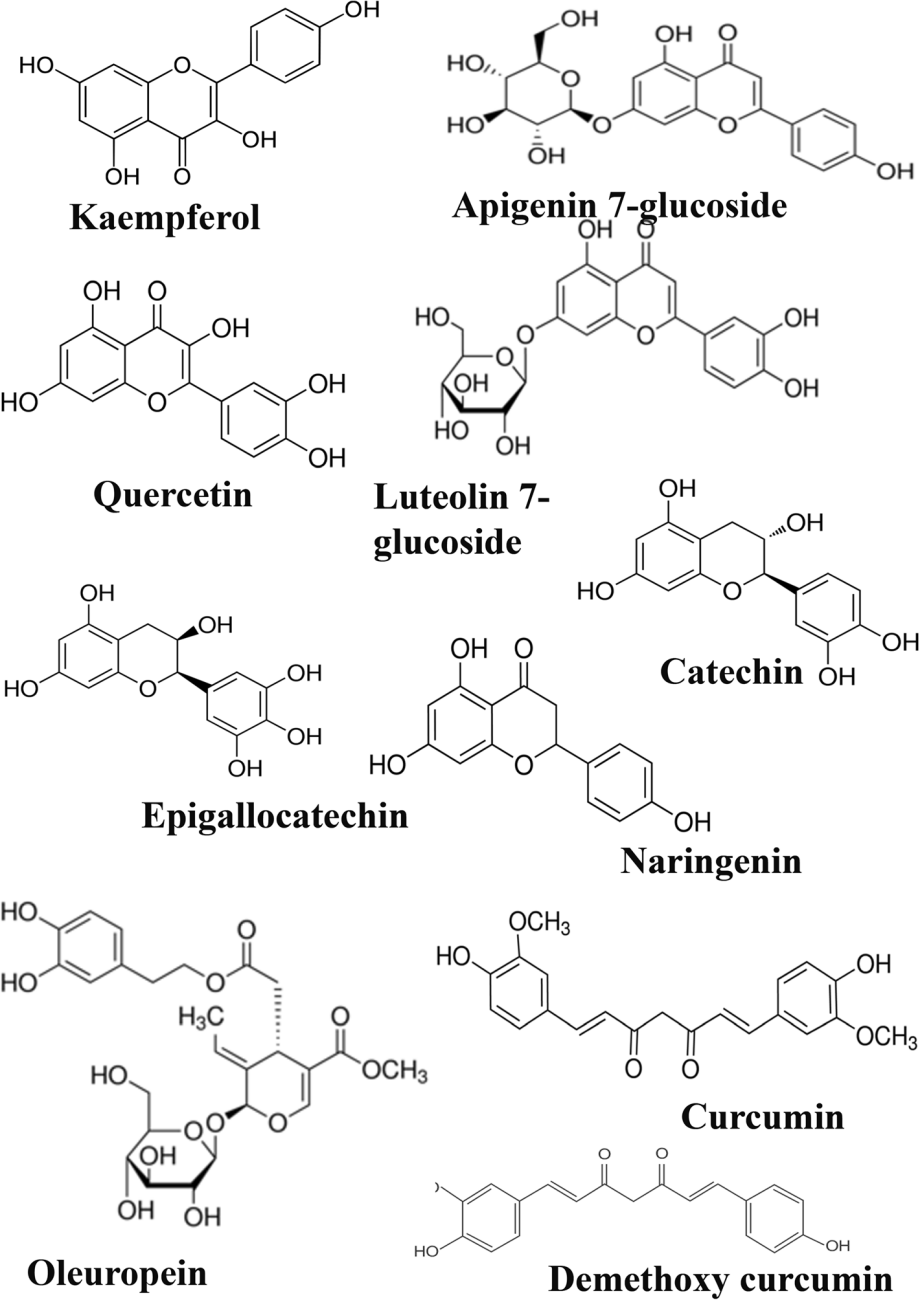
Suggested flavonoid inhibitors of the SARS CoV-2 main protease

The mechanism of coronavirus inhibition by polyphenols includes inhibition of the main protease (M^pro^) or by inhibiting the papain-like protease and signalling pathways. The M^pro^ structure is similar to the isozymes, but there are differences between SARS and SARS-CoV-2. The major polyphenols that are suggested to be M^pro^ inhibitors are explained well in context (Fig. 5). Polyphenols isolated from *Broussonetia papyrifera* have promising effects on the M^pro^ of SARS-CoV and SARS-CoV-2, and the major compounds showing better inhibiting effects are broussochalcone A, papyriflavonol A, 3′-(3-methylbut-2-enyl)-3′,4′,7-trihydroxyflavane, broussoflavan A, kazinol F, and kazinol J. The above compounds were correctly docked at the active site residues (His41 and Cys145) of M^pro^ and exhibited good binding affinity (− 7.6 to − 8.2 kcal/mol) [72]. The tea polyphenols are also suitable inhibitors of the SARS-CoV-2 M^pro^ [73]. Out of the seven polyphenols, epigallocatechin, gallocatechin, catechin, epicatechin, catechin gallate, epigallocatechin gallate, epicatechin gallate, and gallocatechin-3-gallate, only three had a good binding affinity. They are epigallocatechin gallate, epicatechin gallate, and gallocatechin-3-gallate. These three molecules formed more numbers of hydrogen bonds with the active site. They interacted with the residues such as His41 and Cys145, and also, non-covalent, polar, and hydrophobic interactions stabilized the complexes. The interaction pattern displayed by other polyphenols (epigallocatechin, gallocatechin, catechin, epicatechin, and catechin gallate) was in somewhat divergent binding mode. The epigallocatechin–M^pro^ complex had two polar contacts with Ser46 and Asn142, four hydrophobic interactions with Leu141, Met165, Glu166, and Ala191, and four hydrogen bonds with Ser144 (2.5 Å), His163 (2.9 Å & 3.3 Å), and Gln192 (2.6 Å). The binding of the three polyphenols (epigallocatechin gallate, epicatechingallate, and gallocatechin-3-gallate) ranged between − 7.6 and − 9.0 kcal/mol with the lowest affinity for gallocatechin-3-gallate and the highest affinity for epigallocatechin gallate. Oolonghomobisflavan-A, theasinensin-D, and theaflavin-3-O-gallate compounds from the tea plant also showed high inhibitory potentials against M^pro^ [74].

Andrographolide from *Andrographis paniculata* was found to be an inhibitor of M^pro^ [75]. Molecular docking, molecular dynamics simulation, and binding energy studies conformed to the earlier data on the binding affinity. The role of ursolic acid, carvacrol, and oleanolic acid against M^pro^ was investigated [32]. Carvacrol had lesser binding energy compared to the other two molecules, but the mode of interaction is rationally sound. The stability studies showed that the ligand–protein complexes were stable at 50-ns dynamics simulation. The inhibitors fulfilled the ADME parameters as well as Lipinski’s rule of five also. The bioactive molecules from Indian spices were used for the studies against M^pro^. The compounds were retrieved from the Zinc database, and the results expressed that carnosol revealed the highest binding affinity of − 8.2 kcal/mol. Arjunglucoside-I (− 7.88 kcal/mol) and rosmanol (− 7.99 kcal/mol) also showed a strong and stable binding affinity with good ADME properties. MD simulations of these compounds at 50 ns showed strong hydrogen-bonding interactions with the protein active site and endured stability inside the active site [76].

Computational methods also proved the effect of luteolin against SARS CoV-2. Luteolin bound to the active site of M^pro^ with low binding energy than the control molecule chloroquine [69]. The antioxidant molecule quercetin also has a similar effect. The effect of quercetin on SARS CoV has already been reported [77] and its effect on SARS CoV-2 has been studied. It showed a better binding affinity towards the spike protein, ACE2, RdRp, and PLpro than the M^pro^ (− 5.6 kcal/mol), signifying good potential against SARS-CoV-2 [17]. Further, in silico studies showed that flavonoid 5,7-dimethoxyflavanone-4′-O-β-d-glucopyranoside and terpenoid caesalmin B and bonducellpin D also have a greater binding affinity towards M^pro^ of both SARS-CoV-1 and SARS-CoV-2. Compared to the standard drug α-ketoamide, the binding energy of these compounds ranged from −8 to −11 kcal/mol [78]. Additional data indicated the effect of common polyphenols like naringenin, apigenin-7-glucoside, luteolin-7-glucoside, demethoxycurcumin, curcumin, oleuropein, kaempferol, quercetin, and epigallocatechin against SARS CoV-2 and the results were supported by molecular docking studies [79]. The anti-tussive agents like myricitrin and δ-viniferin shared similar inhibitory pattern towards M^pro^ [80]. A common symptom of COVID-19 is cough and the screenings of about 82 molecules from Ayurvedic anti-tussive molecules were carried out against SARS-CoV-2. From these, myricitrin, chrysanthemin, and δ-viniferin displayed significant inhibitory activity and δ-viniferin showed binding energy of − 8.4 kcal/mol, myricitrin with − 8.9 kcal/mol and ligand-protein interactions were stabilized by hydrogen bonding, Pi–Pi interaction, van der Waals forces, and hydrophobic interactions. Flavonoids from *Cephalotaxus wilsoniana*, Taiwan homoflavone A, also had a binding affinity of about − 9.6 kcal/mol and the ADME studies indicated that the compound could be a promising drug lead molecule. The bound form of M^pro^ with Taiwan homoflavone A is stabilized by hydrogen bond with Thr199 and electrostatic interaction with Leu189. Similarly, the compound isolated from *Lactuca virosa* also has an inhibitory effect towards M^pro^. Lactucopirin 15-oxolate, the derivative of lactucin and oxalic acid complex with M^pro^ at the active site residues such as Ser144, Gly143, and Cys145 have a binding energy of − 8.2 kcal/mol. Likewise, anti-HIV and anti-influenza drugs were repurposed against M^pro^, and about seventeen molecules from these categories were also screened. Among them, a macrocyclic derivative Nympholide A, from an aquatic plant of the Nymphaeaceae family, also had binding energy of − 7.8 kcal/mol and formed hydrogen bond interactions with Met165, Glu166, Leu167 [80].

Similar to flavonoids (Fig. 5), terpenoids also are having a prominent effect on viral disease, and the preliminary results indicate the use of these compounds as drug leads. The general subclass of terpenoids, mainly the mono and sesquiterpenoids include (*E*)-nerolidol, (*E*)-β-farnesene, geraniol, and linalool also showed a free energy change of − 26.44 kcal/mol, − 27.56, − 24.71, and − 24.05 kcal/mol respectively against M^pro^. The presence of these molecules in plants like *Copaifera* sp., *Zingiber officinale*, *Matricaria recutita, Cinnamomum zeylanicum*, *Citrus reshni*, *Ocimum basilicum*, *Melissa officinalis*, *Cymbopogon citratus*, *Lavandula angustifolia*, *Pelargonium graveolens* are confirmed [81]. In addition to these, other essential oils like eucalyptus oil and eucalyptol are also used against respiratory disease symptoms. Its effect as inhibiting to SARC-CoV-2 using in vitro assays and molecular docking techniques were reported. The prominent constituents of eucalyptus oil are jensenone and 1,8-cineole, and their effect towards M^pro^ was elucidated, and they interacted with active site residues through hydrophobic interaction, hydrogen bond, and ionic interactions. 1,8-cineole is pharmacologically more critical because it is the main ingredient of pharmaceutical products that affect respiratory ailments when inhaled as vapours in warm water or applied on the nose [82, 83]. The studies on bronchial asthma patients using 1,8-cineole showed a 36% reduction in the use of steroids. The compound has broad-spectrum activity against asthma, COPD, bronchial inflammations [84]. Hence, the effect of this compound on respiratory ailments could also be a choice as a drug lead against SARS-CoV-2.

Furthermore, SARS-CoV-2 M^pro^ inhibitors were also screened from the marine natural databases. MDS studies were carried out with Pharmit (N3/SARS-CoV-2 M^pro^) and Marine Natural Product (MNP) library data. The experiments revealed seventeen active molecules from the data library. The inhibitory results were obtained for the compounds belonging to the category of pseudo-peptides, flavonoids, and floronates. The screened data showed the prevalent natural molecule that inhibited SARS M^pro^ was an oligomer of phloroglucin (1,3,5-trihydroxybenzene) derived from brown algae *Sargassum spinuligerum*. Whereas, the compound that inhibited SARS-CoV-2 M^pro^ belonged to the florotannin group (8,8′-Bieckol, 6,6′-Bieckol, Dieckol), sequestered from the brown algae *Ecklonia cava* [85, 86].

### Role of traditional medicine against SARS CoV-2

#### Traditional medicine and SARS CoV-2: Ayurveda

The Ayurveda is the Indian traditional system of Medicine with the oldest treatment in history [87–90]. It has many herbal drugs which are referenced/used for various ailments. Studies have shown their antiviral properties. Out of the 56 herbal drugs referenced which suit to treat viral infections, 10 are found to have antiviral activity against viruses such as herpes simplex virus (HSV types 1 and 2), dengue virus (DNGV), Newcastle disease virus (NDV), Sindbis virus (SINV), measles virus, and poliovirus [91]. The ten plants mentioned, include *Withania somnifera, Tinospora cordifolia*, and *Ocimum sanctum.* Molecular docking and simulation studies of the withanoside V and somniferine from *Withania somnifera* (Ashwagandha), tinocordiside from *Tinospora cordifolia,* vicenin, isorientin, and 4′-O-glucoside 2″-O-p-hydroxybenzoagte from *Ocimum sanctum* showed good inhibitory potential towards M^pro^ of SARS CoV-2 [92]. In another report, the compounds from *Tinospora cordifolia* showed similar results against M^pro^ [93]. The compounds selected from *Tinospora cordifolia* included^β^-sitosterol, coline, octacosanol, tetrahydropalmatine, and berberine. The results of the study showed that berberine is the most significant compound having interaction with M^pro^ [93]. The phytochemicals present in the rasayanik herbs [94] are methyl eugenol, oleanolic acid, ursolic acid, withanone, withanolides, tinocordiside. The SARS-CoV-2 inhibitory effect of Indian ginseng was proven in silico by Chikhale et al. [95]. The matrix metalloproteinase inhibitors identified from *Camellia sinensis* showed M^pro^ inhibition [96]. The computational studies proved that the bioactive compounds had a significant effect on SARS CoV-2. There is more literature based on in silico studies of phytocompounds of herbs used in Ayurvedic medicines to manage influenza-like symptoms [88, 97–99].

#### Traditional medicine and SARS CoV-2: Siddha

Siddha is a traditional system of Medicine in India, an offshoot of Ayurveda. It is herbal, mineral, and combinations of both. The distinct herbal formulations prepared in this system have applications in various ailments and symptoms. During the time of the dengue outbreak in India, the herbal preparation *Nilavembu Kudineer* was used to control and prevent the disease. If left untreated at an early stage, it may lead to chronic respiratory deficiency known as *Sanni* (*influenza*) [100]/*Kabasura Kudineer Chooranam*, and a novel formulation JACOM used in the Siddha medicine [101] is made from fifteen plants used to treat influenza and related disorders. The compounds of the above formulations were analysed in silico as inhibitors of the COVID-19 spike protein. Molecular docking studies were carried out with the molecules present in the plants as the ligands and the spike glycoprotein (PDB ID: 6VSB) as the target. The compounds selected for the study included scutellarein, magnoflorine, cycleanine, cyperene, β-selinene, 6-methoxygenkwanin, luteolin, costunolide, elemol, tinosponone, bharangin, β-sesquiphellandrene, β-bisabolene, geranial, piperine, piperlonguminine, eugenol, β-caryophyllene, stigmosterol, 3-(2,4-dimethoxyphenyl)-6,7-dimethoxy-2,3-dihydrochromen-4-one, squalene, γ-sitosterol, andrograpanin, 5-hydroxy-7,8-dimethoxyflavanone, lupeol, betulin, chebulagic acid, gallic acid, vasicinone, carvacrol, cirsimaritin, and chrysoeriol from choornam and quercetin, meliacine, vasicine, andrographolide, and ursolic acid from JACOM [102]. Molecular docking studies were carried out with RBD of the S protein and the molecules of the plants used to make the formulation mentioned above. The binding affinity results showed that quercetin, luteolin, and chrysoeriol firmly bound with the RBD domain with a binding score of − 11.159, − 11.392, and − 11.478 kcal/mol respectively, as reported elsewhere in this article [102].

The nine phytoconstituents (6 plants) were found to be the best lead and drug candidates with good synthetic accessibility for production. The best-ranked molecules include quercetin (− 11.47), luteolin (− 11.15), chrysoeriol (− 11.39), magnoflorine (− 9.76), 6-methoxygenkwanin (− 9.293), cirsimaritin (− 9.22), 5-hydroxy-7,8-dimethoxyflavanone (− 9.03), tinosponone (− 8.14), vasicinone (− 8.16) and the plants in which these compounds are present were further chosen, and a new formulation was made, known as “SNACK-V” formulation. Thus, a new formulation was made of the plants which showed compound/s with higher binding affinity. The plants which contain the high score compounds are *Plectranthus ambonicus*, *Tinospora Cordifolia*, *Sida acuta*, *Adhatoda vasica*, *Andrographis paniculata*, and *Costus speciosus*, and the formulation was prepared. This was recommended for further study for their properties of immunomodulation, expectorant, and antipyretic and thereby restoring the normal health [102]*.* The results of this endeavour are yet to be made available.

#### Traditional medicine and SARS CoV-2: TCM

In the current scenario of pandemic and also in the previous outbreak in 2003 different preparations from Traditional Chinese Medicine were used. The medicinal preparations used for fever and fatigue are Shufeng Jiedu capsules, Lianhua Qingwen capsules, and Jinhua Qinggan granules, and for gastrointestinal discomfort, Huoxiang Zhengqi capsules can be used as recommended by TCM. The main ingredient of Lianhua Qingwen is licorice, and the main component is glycyrrhizic acid which is an excellent antiviral agent [68, 69]. Its antiviral activity against SARS CoV is proven earlier [103]. The preparations selected for the trials in China include Qing Yi-4, Guan-2 Formula*,* Guan-2 Formula Tan Re Qing Injection, and Lian Hua Qing Wen. As per the National Health Commission (NHC) of the People’s Republic of China, the herbal preparations that could be used to treat patients within the medical observation period, probably as a protective measure, are Huo Xiang Zheng Qi Shui, Lian Hua Qing Wen Capsule, Shu Feng Jie Du Capsule, and Jin Hua Qing Gan Granule. In the clinical treatment period, Qing Fei Pai Du Tang, Xi Yan Ping Injection, Xue Bi Jing injection, Re Du Ning Injection, Tan Re Qing Injection, and Xing Nao Jing could be used. Moreover, for the patients in chronic condition, Shen Fu Injection, Sheng Mai Injection, Shen Mai Injection, Su He Xiang Pill, and An Gong Niu Huang Pill should be administered [104]*.* In addition to this, the other Chinese herbs used to treat COVID-19 are *Astragalus membranaceus, Glycyrrhizae uralensis, Saposhnikoviae divaricata, Rhizoma Atractylodis Macrocephalae, Lonicerae Japonicae Flos, Fructus forsythia, Atractylodis Rhizoma, Radix platycodonis, Agastache rugosa*, and *Cyrtomium fortune J. Sm* [105]*.* There are more literature based on in silico studies of phytocompounds of herbs used in TCM to manage influenza-like symptoms [30, 104, 106–111].

### Free fatty acids as SARS CoV-2 spike protein inhibitors

SARS-CoV-2 spike protein–linoleic acid complex was formed during the preparation of the protein sample for its cryo-EM structure determination [1]. It showed that linoleic acid has an affinity for the SARS-CoV-2 S protein and the ligand binding might render the S protein defective for the host membrane binding, leading to viral disease inhibition. It is discerned from the above that fatty acids may be SARS-CoV-2 inhibitors, available from vegetable sources, and it may be found that oils and fats from vegetable and animal sources are used in traditional medicines, more importantly in the Ayurvedic system. Hence, capric, caprylic, lauric, linoleic, myristic, oleic, palmitic, and stearic acids (Table 1) were selected for checking free fatty acid: SARS-CoV-2 spike protein interaction study to supplement the observation reported by Toelzer et al. (2020).

**Table 1:**
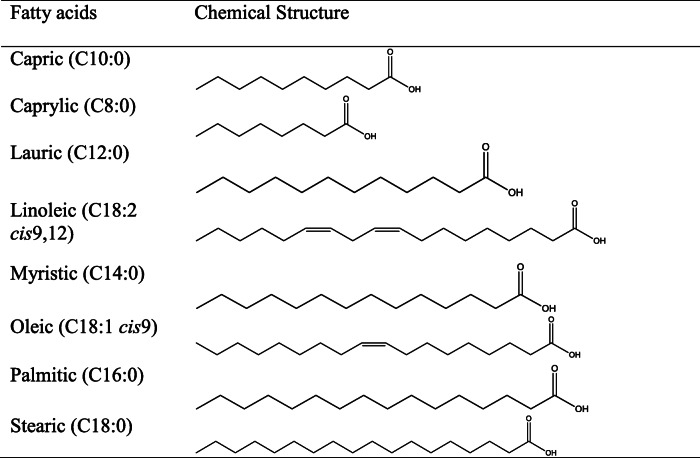
The fatty acids selected for the studies

#### Protein preparation, receptor grid generation and ligand preparation

The cryo-electron microscopy (EM) model of the SARS-CoV-2 spike protein (PDB ID: 6VXX) was downloaded from PDB. Downloaded protein was prepared using the protein preparation wizard in the Maestro software (Maestro, v11.4, Schrodinger, LLC, NewYork, NY). The cryo-EM structure of the spike protein is in prefusion conformation with a resolution of 3.5 Å. The observed structure of the spike protein has both up and down conformations, which means the receptor’s inaccessible state and the receptor’s accessible state, respectively. In the present study, the whole protein was selected, and protein preparation was carried out. In the pre-processing step in protein preparation wizard, the hetero group having bond order and formal charges were added, hydrogen atom added to all the atoms in the system, disulphide bond and zero-order bonds to metals were created, and water molecules within 5 Å in the hetero groups were removed. After pre-processing, the structure refinement step was followed in which missing side-chain atoms were incorporated and the whole trimer was modelled [112]. Then, for each structure, a brief relaxation was performed with Impact Refinement module (Impref) where all atom-constrained minimization was carried out with the aid of force field OPLS_2005 to relieve steric clashes present in the original PDB structure [113]. After reaching the RMSD cut-off of 0.30 Å, the minimization job achieved termination. A free fatty acid–binding pocket was identified by Toelzer et al. [1] in the structure of the spike glycoprotein with a hydrophobic area having a greasy, tube-like appearance. The amino acids (Cys336, Cys361, Val341, Phe342, Phe374, Phe377, Phe392, Cys379–Cys432, Arg408, Gln409, and Trp436) in this area were selected as the binding cavity, and a grid box was prepared around this and receptor grid generation was performed by the receptor grid generation panel in the GLIDE module of Maestro [114]. The ligands for screening, shown above, were selected from the PUBCHEM database. Onset to screening, the ligands were prepared using the LigPrep module with Epik to expand protonation and tautomeric states at neutral pH (7±1). The energy minimization of ligands also was carried out with the OPLS_2005 force field.

#### Induced fit docking

The molecules showing good Glide XP scores in preliminary docking trials were taken for flexible docking. The induced fit docking (IFD) protocol (Maestro package version 11.4 from Schrödinger, LLC) available within the Schrödinger suite was employed [115]. Briefly, this protocol involved docking the ligand using a softened potential, and refining selected docked poses using Prime side-chain prediction and minimization. The refined protein conformations are then used for the final Glide docking step, where ligands are re-docked, keeping the protein rigid. Default values were used for all Glide and Prime parameters. As the protein was prepared in advance, no additional refinement was performed at this stage. For the initial Glide docking, both the receptor and ligand van der Waals scaling were set to 0.50. Up to 20 poses were kept. The Prime induced-fit step refined residues within 5.0 Å of the ligand poses by optimizing their side chains. In the final step, the ligand poses were re-docked using Glide SP into structures within 30.0 kcal/mol of the top 20 structures. The docked pose was compared with the linoleic acid docking structure based on the earlier report [1]. The glide score obtained for this docking was used as a reference value, to which the G scores obtained for the docked compounds were compared.

#### Calculation of free binding energy (MM-GBSA)

The binding free energy studies were carried out for the best hits obtained from XP docking. MM-GBSA in the prime module of the Schrodinger suite was used for this study [116]. For the calculation of binding free energy, this method combines molecular mechanics energies (EMM), surface generalized born solvation model for polar solvation (GSGB), and a nonpolar solvation term (GNP) between the protein and ligand molecule. The term GNP includes nonpolar solvent accessible surface area and van der Waals interactions. The binding free energy was calculated using the following equation.

$$ \Delta  {G}_{Bind}={G}_{Complex}-\left({G}_{Protein}+{G}_{Ligand}\right) $$

where

G = EMM + GSGB + GNP

Since the computational studies are promising for identifying the drug leads within a short period, the molecular docking may determine the effect of ligands towards the target protein molecule. The binding affinity of the ligand towards proteins is stabilized by various interactions like hydrogen bonding, hydrophobic interactions, ionic interaction, π–π bonding, and van der Waals contacts. Similarly, binding energy determination helps to identify and compare the binding affinity of protein and ligand, where the stronger the affinity, the higher is the binding energy. The S of SARS-CoV-2 is the antigenic part which induces an immune response in host cells. The spike protein is responsible for viral attachment, viral fusion, and entry into the host cell through interaction with the ACE 2 receptor.

The structure of SARS-CoV-2 S is explained through cryo-EM and represents mainly two subunits S1 and S2. As described above, the study by Toelzer et al. (2020) showed a free fatty acid-binding pocket in the locked structure of SARS-CoV-2 S. In their experiment, they produced a 2.85-Å cryo-EM structure of SARS-CoV-2 S glycoprotein and disclosed that the RBD strongly binds the essential free fatty acid (FFA), linoleic acid (LA), at the three fused binding pockets. The in vitro analysis showed that this binding stabilized the locked S conformation and thereby reduced interaction with the ACE 2 receptor. This binding pocket is also found both in SARS and MERS-CoV. The experimental evidence suggested that supplementation of linoleic acid with remdesivir subduing SARS-CoV-2 replication in human cells [1]. However, the in vitro results should be checked in vivo for their applicability.

The free fatty acid–binding pocket is a greasy, hydrophobic pocket rich in phenylalanine residues, having a tube-like shape. The carboxyl groups are anchored by arginine and glutamine residues (Arg408 and Gln409) from the adjacent RBD in the trimer, giving rise to a composite LA-binding site and this pocket is only found in the RBD of the trimer. The linoleic acid–binding pocket is surrounded by the following residues: Cys336–Cys361, Val341, Phe342, Phe374, Phe377, Phe392, Cys379–Cys432, Arg408, Gln409, and Trp436. The molecular simulation studies of LA-bound spike proteins showed a stable binding with the trimer than the single RBD domain. The superimposed structure of the LA-bound structure with SARS-CoV-2 apo S structures in the closed conformation recognized a gating helix situated directly at the entrance of the binding pocket. Tyr365 and Tyr369 in the gating helix were displaced about 6 Å upon the binding of LA. It resulted in the opening of the pocket. In the case of an apo form, a gap between neighbouring RBDs places the hydrophilic anchor residues ~ 10 Å away from the position of the LA head group, while LA binding resulted in the movement of adjacent RBD in the trimer towards its neighbouring anchor residues Arg408 and Gln409 and locked down on the head group of LA. This compaction gives rise to the locked structure of the spike protein. Hence, the LA-binding pocket would be a good target for small molecule inhibitors which lock the structure in closed form and interfere with the receptor interaction. This milieu previously proved as a drug target in the case of rhinovirus, in which the locking conformation is irreconcilable with receptor binding and these antivirals were successful in human clinical trials

#### Molecular docking: Results

Based on the linoleic acid-binding pocket studies, in silico studies were carried out with the fatty acids listed above against the SARS-CoV-2 S protein. They are listed in Table 1.

During the flexible docking, flexibility was applied for the protein residues in the second phase of re-docking. Initially, the protein was kept rigid and flexible to the ligand using the Prime module, and interaction was studied within 5 Å from the ligand. The glide score obtained for each compound are shown in Table 2.

**Table 2:** The binding energy and glide score obtained in the molecular docking studies

Compounds	Binding energy (kcal/mol)	Glidescore (kcal/mol)
Stearic (C18:0)	**− 63.70**	**− 3.389**
Oleic (C18:1 *cis*9)	**− 47.01**	**− 5.022**
Palmitic (C16:0)	**− 34.64**	**− 2.398**
Lauric (C12:0) NDa	**− 32.55**	**− 1.996**
Capric (C10:0)	**− 32.22**	**− 2.000**
Caprylic (C8:0) NDa	**− 30.76**	**− 2.480**
Linoleic acid	**− 24.36**	**− 1.117**
Myristic (C14:0)	**− 21.49**	**− 2.434**

**Fig. 6:**
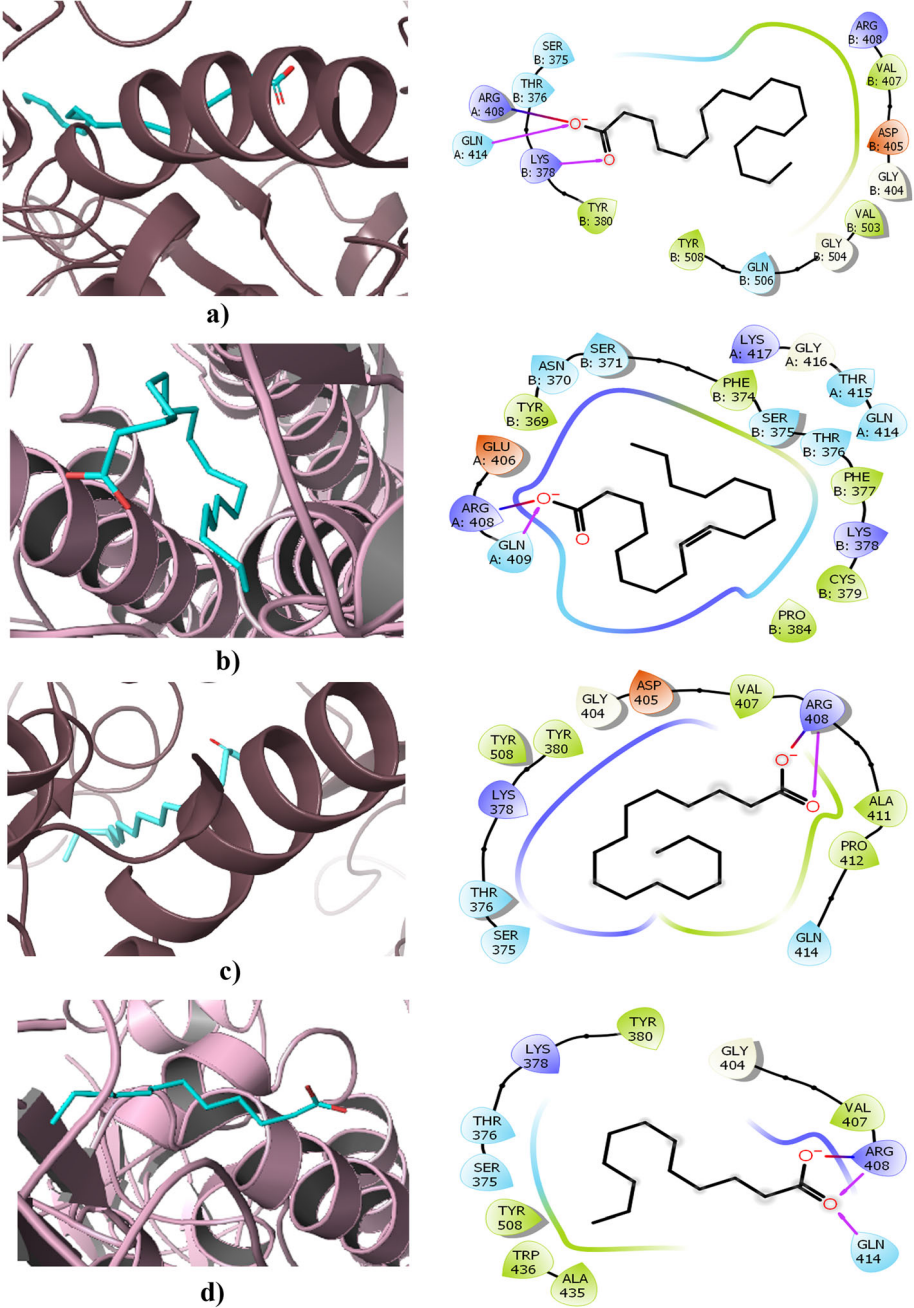
Free fatty acids bound at the binding pocket in the RBD domain of spike proteins **a** stearic acid; **b** oleic acid; **c** palmitic acid; **d** lauric acid

**Fig. 7:**
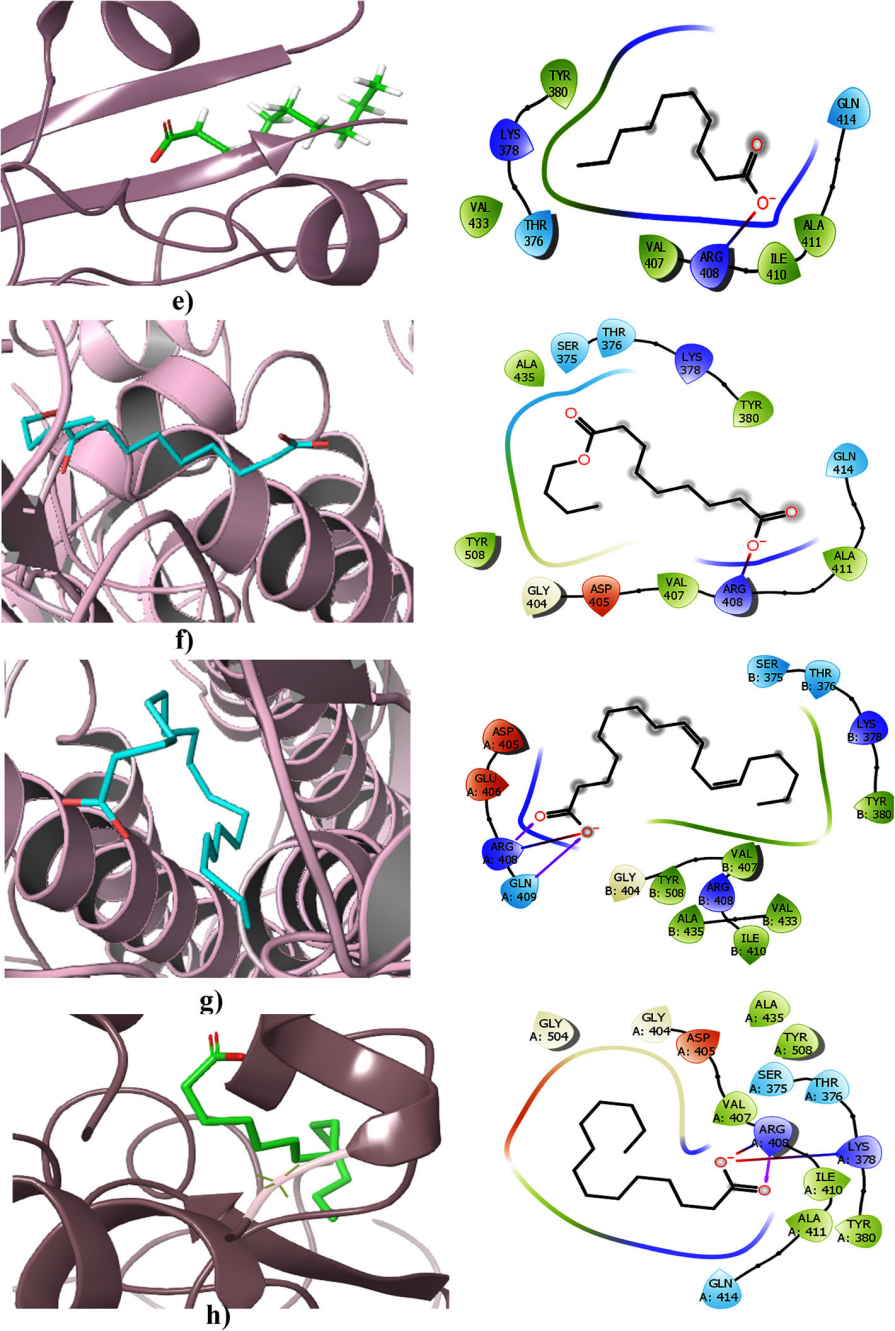
Free fatty acids bound at the binding pocket in the RBD domain of spike proteins **e** capric acid; **f** caprylic acid; **g** linoleic acid; **h** myristic acid

The docking results were compared with the studies carried out by Toelzer and colleagues [1]. In their report, linoleic acid (LA) has been proposed as an inhibitor as inferred from the crystallographic studies of the spike protein. Our results were compared with LA binding. In our in silico study, it was found that LA specifically binds to the site where it interacts with Arg408 and Gln409, as seen in the crystal structure reported with the LA at the same site. The study showed that the LA has strong affinity to S trimer than RBD. Hence, we carried out our study in S trimer, conforming to the reported crystal structure. Cys336–Cys361, Val341, Phe342, Phe374, Phe377, Phe392, Cys379–Cys432, Arg408, Gln409, and Trp436 were also found to be the critical interacting residues in the LA binding pockets of the viral S protein. Nearly all of the selected small molecules, fatty acids analysed exhibited several hydrophobic and hydrogen bond interactions with the main residues of the interface. Analysis of docked complexes revealed that all compounds bind tightly to the binding pocket of the S protein through strong hydrogen bond interactions. Among the all studied compounds, based on binding affinity, stearic acid and oleic acid were found to be the top-ranked compounds. However, all the FAs seem to be prospective in vivo inhibitors of the SARS-CoV-2 S protein. The above is suggestive of the applications of vegetable oils as inhibitors of the two-dimensional interaction maps of selected conformations highlighted the network of hydrophobic as well as hydrogen bond interactions for two ligand interfaces, as shown in Figs. 6 and 7. It may be understood that the fatty acid molecules are interacting mainly with the Arg408. As in the report by Toelzer et al. (2020), Arg408 anchored the carboxyl end of the fatty acid molecules. Thus, the gating helix would open the pocket and lead to the closure of S conformation. The binding energy of stearic acid with RBD of the spike protein was found to be **−** 63.70 kcal/mol, and the complex stabilized by hydrogen bonds with Arg408 and Lys378, and also a salt bridge was formed with Gln414. A similar bonding pattern was identified with all fatty acid molecules tested. Oleic acid formed a hydrogen bond with Gln409 and salt bridge with Arg408 and the binding energy was found to be **−** 47.01 kcal/mol. Palmitic acid formed a hydrogen bond with Arg408. Lauric acid also formed a hydrogen bond with Arg408, and the binding energy was found to be **−** 32.55 kcal/mol. Capric and caprylic acid also shared the same interaction pattern of the hydrogen bond, with Arg408 and salt bridge formation with an appropriate residue. The binding energy was **−** 32.22 and **−** 30.76 kcal/mol, respectively. Myristic acid also shared the same bonding with Arg408 and the binding energy found to be **−** 21.49 kcal/mol. Linoleic acid was docked at this binding pocket and found that the carboxyl group bound with Arg408 and Gln409 through hydrogen bonding. This study validated our hypothesis of SARS-CoV-2:FA interaction, and it further supports the pioneering study reported by Toelzer et al. (2020), where similar observation was made experimentally.

## Conclusion

The above effort to review the recent attempts to assess the studies on remedies for COVID-19 infection has brought out specific exciting facts. Hopefully, this may be of application in the future. The main points are 1. Strategies, an alternative to vaccine development, to treat COVID-19 or similar infections may be of great value provided the insight gained is applied. 2. There are many prospective drug lead compounds in the traditional medicines used for treating influenza or similar symptoms. 3. Though the drug-likeness of certain prospective drug leads compounds are assessed in silico. Their actual usefulness needs to be assessed in vivo. 4. The most striking observation done indirectly in testing the drug-likeness of the prospective drug-lead phytochemicals is the linoleic acid binding onto the SARS-CoV-2 S protein, evidencing the strong possibility of viral inhibition by such compounds. 5. The above conclusion suggests that free fatty acids or their derivatives may occupy a central place and lead role in the drug discovery process for the COVID-19 infection.

The review conducted showed that the possibility of many phytochemicals might be useful as lead compounds for developing drugs to tackle the COVID-19 pandemic. However, the confidence in depending on the in silico results as final would be less compared to thorough in vitro investigations. Hence, the outcome of the present review is the recommendation for thorough MDS analyses of the promising candidate drug leads. Such analyses may be considered together with the application of other in silico methods of prediction of pharmacological properties directing towards the sites of drug-receptor regulation. Also, the present analysis would help formulate new recipes for complementary medicines.

## Data Availability

Data and material are available upon request.

## Abbreviations

SARS: Severe acute respiratory syndrome
MERS: Middle East respiratory syndrome
WHO: World Health Organization
SARS-CoV-2: Severe acute respiratory syndrome coronavirus-2
SARS-HCoV: Severe acute respiratory syndrome human coronavirus
HCoV-OC43: Human coronavirus-OC43
MERS-CoV: Middle East respiratory syndrome coronavirus
ARDS: Acute respiratory distress syndrome
ICU: Intensive care unit
IP10: Interferon-γ (IFN-γ)-induced protein 10
IL12: Interleukin 12
IL1B: Interleukin 1 beta
IL6: Interleukin 6
IFNγ: Interferon-γ
IL15: Interleukin 5
IL17: Interleukin 17
MCP1: Monocyte chemo attractant protein-1
TNFα: Tumour necrosis factor alpha
GCSF: Granulocyte colony-stimulating factor
MIP1α: Macrophage inflammatory protein-1α
CD4^+^ T: Cluster of differentiation 4 T cells
CD8^+^ T: Cluster of differentiation 8 T cells
HLH: Haemophagocytic lymphohistiocytosis
RNA: Ribonucleic acid
ORF1a/ORF1b: Open reading frame 1a/1b
Nsp: Non-structural protein
M^pro^: Main protease
3CL^pro^: 3C-like protease
PL^pro^: Papain-like protease
BatCoV-RaTG13: Bat coronavirus-RaTG13
ACE 2: Angiotensin-converting enzymes 2
TM: Transmembrane
TMPRSS2: Transmembrane (TM) protease serine 2
RBD: Receptor-binding domain
HR1: Heptapeptide repeat
SARS-CoV-2-CTD: Severe acute respiratory syndrome coronavirus-2 C-terminal domain
pp1a and pp1ab: polyproteins 1a and 1ab
HIV: Human immunodeficiency virus
HCV: Hepatitis C virus
RdRp: RNA-dependent RNA polymerase
HCQ: Hydroxychloroquine
GC-MS: Gas chromatography–mass spectrometry
ADME: Absorption, distribution, metabolism, excretion
MD: Molecular dynamics
COPD: Chronic obstructive pulmonary disease
MNP: Marine natural product
HSV: Herpes simplex virus
DNGV: Dengue virus
NDV: Newcastle disease virus
SINV: Sindbis virus
TCM: Traditional Chinese Medicine
RMSD: Root mean square deviation
XP: Extra precision
SP: Standard precision
MM-GBSA: Molecular mechanics energies combined with generalized born and surface area
LA: Linoleic acid

## References

[1] Toelzer C, Gupta K, Yadav SKN, Borucu U, Davidson AD, Williamson MK, Shoemark DK, Garzoni F, Staufer O, Milligan R, Capin J, Mulholland AJ, Spatz J, Fitzgerald D, Berger I, Schaffitzel C (2020). Free fatty acid binding pocket in the locked structure of SARS-CoV-2 spike protein. Science..

[2] Rodriguez-Morales AJ, Cardona-Ospina JA, Gutiérrez-Ocampo E, Villamizar-Peña R, Holguin-Rivera Y, Escalera-Antezana JP, Alvarado-Arnez LE, Bonilla-Aldana DK, Franco-Paredes C, Henao-Martinez AF, Paniz-Mondolfi A, Lagos-Grisales GJ, Ramírez-Vallejo E, Suárez JA, Zambrano LI, Villamil-Gómez WE, Balbin-Ramon GJ, Rabaan AA, Harapan H, Dhama K, Nishiura H, Kataoka H, Ahmad T, Sah R (2020). Clinical, laboratory and imaging features of COVID-19: a systematic review and meta-analysis. Travel Med Infect Dis.

[3] Harapan H, Itoh N, Yufika A, Winardi W, Keam S, Te H, Megawati D, Hayati Z, Wagner AL, Mudatsir M (2020). Coronavirus disease 2019 (COVID-19): a literature review. J Infect Public Health.

[4] Wang C, Horby PW, Hayden FG, Gao GF (2020). A novel coronavirus outbreak of global health concern. Lancet.

[5] Huang C, Wang Y, Li X, Ren L, Zhao J, Hu Y, Zhang L, Fan G, Xu J, Gu X, Cheng Z, Yu T, Xia J, Wei Y, Wu W, Xie X, Yin W, Li H, Liu M, Xiao Y, Gao H, Guo L, Xie J, Wang G, Jiang R, Gao Z, Jin Q, Wang J, Cao B (2020). Clinical features of patients infected with 2019 novel coronavirus in Wuhan, China. Lancet.

[6] Indolfi C, Spaccarotella C (2020). The outbreak of COVID-19 in Italy: fighting the pandemic. J Am Coll Cardiol Case Rep.

[7] WHO Coronavirus Disease (COVID-19) Dashboard. https://covid19.who.int. Accessed 21 May 2021.

[8] Wong CK, Lam CWK, Wu AKL, Ip WK, Lee NLS, Chan IHS, Lit LCW, Hui DSC, Chan MHM, Chung SSC, Sung JJY (2004). Plasma inflammatory cytokines and chemokines in severe acute respiratory syndrome. Clin Exp Immunol.

[9] Assiri A, Al-Tawfiq JA, Al-Rabeeah AA, Al-Rabiah FA, Al-Hajjar S, Al-Barrak A, Flemban H, Al-Nassir WN, Balkhy HH, Al-Hakeem RF, Makhdoom HQ, Zumla AI, Memish ZA (2013). Epidemiological, demographic, and clinical characteristics of 47 cases of Middle East respiratory syndrome coronavirus disease from Saudi Arabia: a descriptive study. Lancet Infect Dis.

[10] Mahallawi WH, Zhang Q, Makhdoum HM, Suliman BA, Khabour OF (2018). MERS-CoV infection in humans is associated with a pro-inflammatory Th1 and Th17 cytokine profile. Cytokine.

[11] Farquhar JW, Claireaux AE (1952). Familial haemophagocytic reticulosis. Arch Dis Child.

[12] Zhong J, Tang J, Ye C, Dong L (2020). The immunology of COVID-19: is immune modulation an option for treatment?. Lancet Rheumatol.

[13] Mehta P, McAuley DF, Brown M, Sanchez E, Tattersall RS, Manson JJ (2020). COVID-19: consider cytokine storm syndromes and immunosuppression. Lancet.

[14] Ruscitti P, Berardicurti O, Iagnocco A, Giacomelli R (2020). Cytokine storm syndrome in severe COVID-19. Autoimmun Rev.

[15] Wu F, Zhao S, Yu B, Chen Y-M, Wang W, Song Z-G, Hu Y, Tao Z-W, Tian J-H, Pei Y-Y, Yuan M-L, Zhang Y-L, Dai F-H, Liu Y, Wang Q-M, Zheng J-J, Xu L, Holmes EC, Zhang Y-Z (2020). A new coronavirus associated with human respiratory disease in China. Nature.

[16] Xu X, Chen P, Wang J, Feng J, Zhou H, Li X, Zhong W, Hao P (2020). Evolution of the novel coronavirus from the ongoing Wuhan outbreak and modeling of its spike protein for risk of human transmission. Sci China Life Sci.

[17] Huang Y, Yang C, Xu X, Xu W, Liu S (2020). Structural and functional properties of SARS-CoV-2 spike protein: potential antivirus drug development for COVID-19. Acta Pharmacol Sin.

[18] Jaimes JA, André NM, Chappie JS, Millet JK, Whittaker GR (2020). Phylogenetic analysis and structural modeling of SARS-CoV-2 spike protein reveals an evolutionary distinct and proteolytically sensitive activation loop. J Mol Biol.

[19] Hu B, Guo H, Zhou P, Shi Z-L (2020). Characteristics of SARS-CoV-2 and COVID-19. Nat Rev Microbiol.

[20] Tiwari R, Dhama K, Sharun K, Yatoo MI, Malik YS, Singh R, Michalak I, Sah R, Bonilla-Aldana DK, Rodriguez-Morales AJ (2020). COVID-19: animals, veterinary and zoonotic links. Vet Q.

[21] Zhou H, Chen X, Hu T, Li J, Song H, Liu Y, Wang P, Liu D, Yang J, Holmes EC, Hughes AC, Bi Y, Shi W (2020). A novel bat coronavirus closely related to SARS-CoV-2 contains natural insertions at the S1/S2 cleavage site of the spike protein. Curr Biol.

[22] Kang CK, Seong M-W, Choi S-J, Kim TS, Choe PG, Song SH, Kim N-J, Park WB, Oh M (2020). *In vitro* activity of lopinavir/ritonavir and hydroxychloroquine against severe acute respiratory syndrome coronavirus 2 at concentrations achievable by usual doses. Korean J Intern Med.

[23] Marzolini C, Stader F, Stoeckle M, Franzeck F, Egli A, Bassetti S, Hollinger A, Osthoff M, Weisser M, Gebhard CE, Baettig V, Geenen J, Khanna N, Tschudin-Sutter S, Mueller D, Hirsch HH, Battegay M, Sendi P (2020) Effect of systemic inflammatory response to SARS-CoV-2 on Lopinavir and hydroxychloroquine plasma concentrations. Antimicrob Agents Chemother 64(9):e01177-20. 10.1128/AAC.01177-20. 10.1128/AAC.01177-20 PMC7449226 32641296

[24] Panagopoulos P, Petrakis V, Panopoulou M, Trypsianis G, Penlioglou T, Pnevmatikos I, Papazoglou D (2020). Lopinavir/ritonavir as a third agent in the antiviral regimen for SARS-CoV-2 infection. J Chemother.

[25] Hashemian SM, Farhadi T, Velayati AA (2020). A review on remdesivir: a possible promising agent for the treatment of COVID-19. Drug Des Dev Ther.

[26] Ko W-C, Rolain J-M, Lee N-Y, Chen P-L, Huang C-T, Lee P-I, Hsueh P-R (2020). Arguments in favour of remdesivir for treating SARS-CoV-2 infections. Int J Antimicrob Agents.

[27] Wang Y-C, Yang W-H, Yang C-S, Hou M-H, Tsai C-L, Chou Y-Z, Hung M-C, Chen Y (2020). Structural basis of SARS-CoV-2 main protease inhibition by a broad-spectrum anti-coronaviral drug. Am J Cancer Res.

[28] Agrawal U, Raju R, Udwadia ZF (2020). Favipiravir: a new and emerging antiviral option in COVID-19. Med J Armed Forces India.

[29] Coomes EA, Haghbayan H (2020). Favipiravir, an antiviral for COVID-19?. J Antimicrob Chemother.

[30] Li L, Wang X, Wang R, Hu Y, Jiang S, Lu X (2020). Antiviral agent therapy optimization in special populations of COVID-19 patients. Drug Des Dev Ther.

[31] Yanai H (2020). Favipiravir: a possible pharmaceutical treatment for COVID-19. J Endocrinol Metab.

[32] Kumar A, Suresh Sharanya C, Jayanandan A, Sadasivan C (2020) Drug repurposing to identify therapeutics against COVID 19 with SARS-Cov-2 spike glycoprotein and main protease as targets: an *in silico* study. 10.26434/chemrxiv.12090408.v1

[33] Uzunova K, Filipova E, Pavlova V, Vekov T (2020). Insights into antiviral mechanisms of remdesivir, lopinavir/ritonavir and chloroquine/hydroxychloroquine affecting the new SARS-CoV-2. Biomed Pharmacother.

[34] Elfiky AA (2020). Anti-HCV, nucleotide inhibitors, repurposing against COVID-19. Life Sci.

[35] Khalili JS, Zhu H, Mak NSA, Yan Y, Zhu Y (2020). Novel coronavirus treatment with ribavirin: groundwork for an evaluation concerning COVID-19. J Med Virol.

[36] Mantlo E, Bukreyeva N, Maruyama J, Paessler S, Huang C (2020). Antiviral activities of type I interferons to SARS-CoV-2 infection. Antivir Res.

[37] Sallard E, Lescure F-X, Yazdanpanah Y, Mentre F, Peiffer-Smadja N (2020). Type 1 interferons as a potential treatment against COVID-19. Antivir Res.

[38] Jianping Z (2020). Randomized, open, blank control study on the efficacy and safety of recombinant human interferon α1β in the treatment of patients with new type of coronavirus infection in Wuhan.

[39] Vincent MJ, Bergeron E, Benjannet S, Erickson BR, Rollin PE, Ksiazek TG, Seidah NG, Nichol ST (2005). Chloroquine is a potent inhibitor of SARS coronavirus infection and spread. Virol J.

[40] Devaux CA, Rolain J-M, Colson P, Raoult D (2020). New insights on the antiviral effects of chloroquine against coronavirus: what to expect for COVID-19?. Int J Antimicrob Agents.

[41] Rebeaud ME, Zores F (2020) SARS-CoV-2 and the use of chloroquine as an antiviral treatment. Front Med 7:184. 10.3389/fmed.2020.00184 10.3389/fmed.2020.00184 PMC7193267 32391371

[42] Acharya Y, Sayed A (2020). Chloroquine and hydroxychloroquine as a repurposed agent against COVID-19: a narrative review. Ther Adv Infect.

[43] Braz HLB, de Silveira JAM, Marinho AD, de Moraes MEA, de Moraes Filho MO, Monteiro HSA, Jorge RJB (2020). *In silico* study of azithromycin, chloroquine and hydroxychloroquine and their potential mechanisms of action against SARS-CoV-2 infection. Int J Antimicrob Agents.

[44] Gentry CA, Humphrey MB, Thind SK, Hendrickson SC, Kurdgelashvili G, Williams RJ (2020). Long-term hydroxychloroquine use in patients with rheumatic conditions and development of SARS-CoV-2 infection: a retrospective cohort study. Lancet Rheumatol.

[45] Singh AK, Singh A, Shaikh A, Singh R, Misra A (2020). Chloroquine and hydroxychloroquine in the treatment of COVID-19 with or without diabetes: a systematic search and a narrative review with a special reference to India and other developing countries. Diabetes Metab Syndr.

[46] Bhuiyan FR, Howlader S, Raihan T, Hasan M (2020) Plants metabolites: possibility of natural therapeutics against the COVID-19 pandemic. Front Med 7:444. 10.3389/fmed.2020.00444 10.3389/fmed.2020.00444 PMC7427128 32850918

[47] Hussain I, Hussain A, Alajmi MF, Rehman MT, Amir S (2021). Impact of repurposed drugs on the symptomatic COVID-19 patients. J Infect Public Health.

[48] Shah RR (2021). Chloroquine and hydroxychloroquine for COVID-19: perspectives on their failure in repurposing. J Clin Pharm Ther.

[49] Kamat S, Kumari M (2021). Repurposing chloroquine against multiple diseases with special attention to SARS-CoV-2 and associated toxicity. Front Pharmacol.

[50] Gorlenko CL, Kiselev HY, Budanova EV, Zamyatnin AA, Ikryannikova LN (2020) Plant secondary metabolites in the battle of drugs and drug-resistant bacteria: new heroes or worse clones of antibiotics? Antibiotics 9(4):170. 10.3390/antibiotics9040170 10.3390/antibiotics9040170 PMC7235868 32290036

[51] Ghildiyal R, Prakash V, Chaudhary VK, Gupta V, Gabrani R, Swamy MK (2020). Phytochemicals as antiviral agents: recent updates. Plant-derived bioactives: production, properties and therapeutic applications.

[52] Srivastav VK, Egbuna C, Tiwari M (2020) Chapter 1 - plant secondary metabolites as lead compounds for the production of potent drugs. In: Egbuna C, Kumar S, Ifemeje JC, Ezzat SM, Kaliyaperumal S (eds) Phytochemicals as lead compounds for new drug discovery. Elsevier, pp 3–14

[53] Achan J, Talisuna AO, Erhart A, Yeka A, Tibenderana JK, Baliraine FN, Rosenthal PJ, D’Alessandro U (2011). Quinine, an old anti-malarial drug in a modern world: role in the treatment of malaria. Malar J.

[54] Liu A-L, Du G-H, Patra AK (2012). Antiviral properties of phytochemicals. Dietary phytochemicals and microbes.

[55] Chernyshov VV, Yarovaya OI, Fadeev DS, Gatilov YV, Esaulkova YL, Muryleva AS, Sinegubova KO, Zarubaev VV, Salakhutdinov NF (2020). Single-stage synthesis of heterocyclic alkaloid-like compounds from (+)-camphoric acid and their antiviral activity. Mol Divers.

[56] Lee JL, Loe MWC, Lee RCH, Chu JJH (2019). Antiviral activity of pinocembrin against Zika virus replication. Antivir Res.

[57] Gómez-Calderón C, Mesa-Castro C, Robledo S, Gómez S, Bolivar-Avila S, Diaz-Castillo F, Martínez-Gutierrez M (2017). Antiviral effect of compounds derived from the seeds of Mammea americana and Tabernaemontana cymosa on dengue and chikungunya virus infections. BMC Complement Altern Med.

[58] Cecílio AB, de Oliveira PC, Caldas S, Campana PRV, Francisco FL, Duarte MGR, de Mendonça LAM, de Almeida VL (2016). Antiviral activity of Myracrodruon urundeuva against rotavirus. Rev Bras.

[59] Lipson SM, Karalis G, Karthikeyan L, Ozen FS, Gordon RE, Ponnala S, Bao J, Samarrai W, Wolfe E (2017). Mechanism of anti-rotavirus synergistic activity by epigallocatechin gallate and a proanthocyanidin-containing nutraceutical. Food Environ Virol.

[60] Anusuya S, Gromiha MM (2019). Structural basis of flavonoids as dengue polymerase inhibitors: insights from QSAR and docking studies. J Biomol Struct Dyn.

[61] Ohemu TL, Agunu A, Chollom SC, Okwori VA, Dalen DG, Olotu PN (2018). Preliminary phytochemical screening and antiviral potential of methanol stem bark extract of Enantia chlorantha Oliver (Annonaceae) and Boswellia dalzielii Hutch (Burseraceae) against Newcastle disease in ovo. Eur J Med Plants.

[62] Fan W, Qian S, Qian P, Li X (2016). Antiviral activity of luteolin against Japanese encephalitis virus. Virus Res.

[63] Ismaeel MYY, Dyari HRE, Yaacob WA, Ibrahim N (2018). *In vitro* antiviral activity of aqueous extract of Phaleria macrocarpa fruit against herpes simplex virus type 1. AIP Conf Proc.

[64] LeCher JC, Diep N, Krug PW, Hilliard JK (2019) Genistein Has antiviral activity against herpes B virus and acts synergistically with antiviral treatments to reduce effective dose. Viruses 11(6):499. 10.3390/v11060499 10.3390/v11060499 PMC6630448 31159175

[65] Pandey P, Rane JS, Chatterjee A, Kumar A, Khan R, Prakash A, Ray S (2020). Targeting SARS-CoV-2 spike protein of COVID-19 with naturally occurring phytochemicals: an *in silico* study for drug development. J Biomol Struct Dyn.

[66] Wahedi HM, Ahmad S, Abbasi SW (2020). Stilbene-based natural compounds as promising drug candidates against COVID-19. J Biomol Struct Dyn.

[67] Kulkarni SA, Nagarajan SK, Ramesh V, Palaniyandi V, Selvam SP, Madhavan T (2020). Computational evaluation of major components from plant essential oils as potent inhibitors of SARS-CoV-2 spike protein. J Mol Struct.

[68] Bailly C, Vergoten G (2020). Glycyrrhizin: an alternative drug for the treatment of COVID-19 infection and the associated respiratory syndrome?. Pharmacol Ther.

[69] Yu R, Chen L, Lan R, Shen R, Li P (2020). Computational screening of antagonists against the SARS-CoV-2 (COVID-19) coronavirus by molecular docking. Int J Antimicrob Agents.

[70] Basu A, Sarkar A, Maulik U (2020). Molecular docking study of potential phytochemicals and their effects on the complex of SARS-CoV2 spike protein and human ACE2. Sci Rep.

[71] Thuy BTP, My TTA, Hai NTT, Hieu LT, Hoa TT, Thi Phuong Loan H, Triet NT, Anh TTV, Quy PT, Tat PV, Hue NV, Quang DT, Trung NT, Tung VT, Huynh LK, Nhung NTA (2020). Investigation into SARS-CoV-2 resistance of compounds in garlic essential oil. ACS Omega.

[72] Park J-Y, Yuk HJ, Ryu HW, Lim SH, Kim KS, Park KH, Ryu YB, Lee WS (2017). Evaluation of polyphenols from Broussonetia papyrifera as coronavirus protease inhibitors. J Enzyme Inhib Med Chem.

[73] Ghosh R, Chakraborty A, Biswas A, Chowdhuri S (2020). Identification of polyphenols from Broussonetia papyrifera as SARS CoV-2 main protease inhibitors using *in silico* docking and molecular dynamics simulation approaches. J Biomol Struct Dyn.

[74] Bhardwaj VK, Singh R, Sharma J, Rajendran V, Purohit R, Kumar S (2020). Identification of bioactive molecules from tea plant as SARS-CoV-2 main protease inhibitors. J Biomol Struct Dyn.

[75] Enmozhi SK, Raja K, Sebastine I, Joseph J (2020). Andrographolide as a potential inhibitor of SARS-CoV-2 main protease: an *in silico* approach. J Biomol Struct Dyn.

[76] Umesh KD, Selvaraj C, Singh SK, Dubey VK (2020). Identification of new anti-nCoV drug chemical compounds from Indian spices exploiting SARS-CoV-2 main protease as target. J Biomol Struct Dyn.

[77] Ryu YB, Jeong HJ, Kim JH, Kim YM, Park JY, Kim D, Naguyen TT, Park SJ, Chang JS, Park KH, Rho MC, Lee WS (2010). Biflavonoids from Torreya nucifera displaying SARS-CoV 3CL(pro) inhibition. Bioorg Med Chem.

[78] Gurung AB, Ali MA, Lee J, Farah MA, Al-Anazi KM (2020). Unravelling lead antiviral phytochemicals for the inhibition of SARS-CoV-2 Mpro enzyme through *in silico* approach. Life Sci.

[79] Khaerunnisa S, Kurniawan H, Awaluddin R, Suhartati S, Soetjipto S (2020). Potential inhibitor of COVID-19 main protease (Mpro) from several medicinal plant compounds by molecular docking study.

[80] Joshi RS, Jagdale SS, Bansode SB, Shankar SS, Tellis MB, Pandya VK, Chugh A, Giri AP, Kulkarni MJ (2020) Discovery of potential multi-target-directed ligands by targeting host-specific SARS-CoV-2 structurally conserved main protease. J Biomol Struct Dyn 39(9):1–16. 10.1080/07391102.2020.1760137 10.1080/07391102.2020.1760137 PMC7212545 32329408

[81] da Silva JKR, Figueiredo PLB, Byler KG, Setzer WN (2020). Essential oils as antiviral agents, potential of essential oils to treat SARS-CoV-2 infection: an in-silico investigation. Int J Mol Sci.

[82] Sharma H, Chauhan P, Singh S (2018). Evaluation of the anti-arthritic activity of Cinnamomum cassia bark extract in experimental models. Integr Med Res.

[83] Asif M, Saleem M, Saadullah M, Yaseen HS, Al Zarzour R (2020). COVID-19 and therapy with essential oils having antiviral, anti-inflammatory, and immunomodulatory properties. Inflammopharmacology.

[84] Juergens LJ, Worth H, Juergens UR (2020). New perspectives for mucolytic, anti-inflammatory and adjunctive therapy with 1,8-cineole in COPD and asthma: review on the new therapeutic approach. Adv Ther.

[85] Chojnacka K, Witek-Krowiak A, Skrzypczak D, Mikula K, Młynarz P (2020). Phytochemicals containing biologically active polyphenols as an effective agent against Covid-19-inducing coronavirus. J Funct Foods.

[86] Gentile D, Patamia V, Scala A, Sciortino MT, Piperno A, Rescifina A (2020). Putative inhibitors of SARS-CoV-2 main protease from a library of marine natural products: a virtual screening and molecular modeling study. Marine Drugs.

[87] Divya M, Vijayakumar S, Chen J, Vaseeharan B, Durán-Lara EF (2020) South Indian medicinal plants can combat deadly viruses along with COVID-19? - a review. Microb Pathog 148:104277. 10.1016/j.micpath.2020.104277 10.1016/j.micpath.2020.104277 PMC7253980 32473390

[88] Maurya DK, Sharma D (2020). Evaluation of traditional ayurvedic preparation for prevention and management of the novel coronavirus (SARS-CoV-2) using molecular docking approach.

[89] Shetty R, Ghosh A, Honavar SG, Khamar P, Sethu S (2020). Therapeutic opportunities to manage COVID-19/SARS-CoV-2 infection: present and future. Indian J Ophthalmol.

[90] Vellingiri B, Jayaramayya K, Iyer M, Narayanasamy A, Govindasamy V, Giridharan B, Ganesan S, Venugopal A, Venkatesan D, Ganesan H, Rajagopalan K, Rahman PKSM, Cho S-G, Kumar NS, Subramaniam MD (2020). COVID-19: a promising cure for the global panic. Sci Total Environ.

[91] Bharat Rathi, Renu Rathi, Pramod Khobragade (2020) Relevance of Ayurveda anti-viral herbal wisdom from the perspective of current researches. Int J Res Pharm Sci 11:175–182. 10.26452/ijrps.v11iSPL1.2346

[92] Shree P, Mishra P, Selvaraj C, Singh SK, Chaube R, Garg N, Tripathi YB (2020). Targeting COVID-19 (SARS-CoV-2) main protease through active phytochemicals of ayurvedic medicinal plants – Withania somnifera (Ashwagandha), Tinospora cordifolia (Giloy) and Ocimum sanctum (Tulsi) – a molecular docking study. J Biomol Struct Dyn.

[93] Chowdhury P (2020). *In silico* investigation of phytoconstituents from Indian medicinal herb ‘Tinospora cordifolia (giloy)’ against SARS-CoV-2 (COVID-19) by molecular dynamics approach. J Biomol Struct Dyn.

[94] Dimri M, Rajwar VS, Kush L (2020). Rasayana drugs promise better anti-Covid-19 medications. Asian J Pharm Res Dev.

[95] Chikhale RV, Gurav SS, Patil RB, Sinha SK, Prasad SK, Shakya A, Shrivastava SK, Gurav NS, Prasad RS (2020). Sars-cov-2 host entry and replication inhibitors from Indian ginseng: an in-silico approach. J Biomol Struct Dyn.

[96] Kanbarkar N, Mishra S (2020). Matrix metalloproteinase inhibitors identified from Camellia sinensis for COVID-19 prophylaxis: an *in silico* approach. Adv Tradit Med.

[97] Parida PK, Paul D, Chakravorty D (2020). Nature to nurture - identifying phytochemicals from Indian medicinal plants as prophylactic medicine by rational screening to be potent against multiple drug targets of SARS-CoV-2.

[98] Renjith MRD, Sankar DM (2020). Scope of phytochemicals in the management of COVID-19. Pharm Res.

[99] Haridas M, Sasidhar V, Nath P, Abhithaj J, Sabu A, Rammanohar P (2021). Compounds of Citrus medica and Zingiber officinale for COVID-19 inhibition: *in silico* evidence for cues from Ayurveda. Future J Pharm Sci.

[100] Neethu D (2017) Anti-inflammatory, antipyretic and antibacterial study of Kabasura kudineer choornam. In: undefined. /paper/Anti-Inflammatory%2C-Antipyretic-and-Antibacterial-of-Neethu/9cdabecc50a2b1a8b5e1375a03108a71ed20a9fb. Accessed 1 Nov 2020

[101] The Siddha Formulary of India (1992). Government of India, Ministry of Health and Family Welfare, Department of Health.

[102] Kiran G, Karthik L, Shree Devi MS, Sathiyarajeswaran P, Kanakavalli K, Kumar KM, Ramesh Kumar D (2020) *In Silico* computational screening of Kabasura kudineer - official Siddha formulation and JACOM against SARS-CoV-2 spike protein. J Ayurveda Integr Med. 10.1016/j.jaim.2020.05.009 10.1016/j.jaim.2020.05.009 PMC7247487 32527713

[103] Cinatl J, Morgenstern B, Bauer G, Chandra P, Rabenau H, Doerr HW (2003). Glycyrrhizin, an active component of liquorice roots, and replication of SARS-associated coronavirus. Lancet.

[104] Yang Y, Islam MS, Wang J, Li Y, Chen X (2020). Traditional Chinese Medicine in the treatment of patients infected with 2019-new coronavirus (SARS-CoV-2): a review and perspective. Int J Biol Sci.

[105] Luo H, Tang Q-L, Shang Y-X, Liang S-B, Yang M, Robinson N, Liu J-P (2020). Can Chinese medicine be used for prevention of corona virus disease 2019 (COVID-19)? A review of historical classics, research evidence and current prevention programs. Chin J Integr Med.

[106] Wei T, Wang H, Wu X, Lu Y, Guan S, Dong F, Dong C, Zhu G, Bao Y, Zhang J, Wang G, Li H (2020). *In Silico* screening of potential spike glycoprotein inhibitors of SARS-CoV-2 with drug repurposing strategy. Chin J Integr Med.

[107] Hussain G (2019). Vishaharayogas in Sahasrayoga: a review. J Drug Deliv Ther.

[108] Fuzimoto AD, Isidoro C (2020). The antiviral and coronavirus-host protein pathways inhibiting properties of herbs and natural compounds - additional weapons in the fight against the COVID-19 pandemic?. J Tradit Complement Med.

[109] Zhang D, Wu K, Zhang X, Deng S, Peng B (2020). *In silico* screening of Chinese herbal medicines with the potential to directly inhibit 2019 novel coronavirus. J Integr Med.

[110] Choudhry N, Zhao X, Xu D, Zanin M, Chen W, Yang Z, Chen J (2020). Chinese therapeutic strategy for fighting COVID-19 and potential small-molecule inhibitors against severe acute respiratory syndrome coronavirus 2 (SARS-CoV-2). J Med Chem.

[111] Pan H-D, Yao X-J, Wang W-Y, Lau H-Y, Liu L (2020). Network pharmacological approach for elucidating the mechanisms of traditional Chinese medicine in treating COVID-19 patients. Pharmacol Res.

[112] Sastry GM, Adzhigirey M, Day T, Annabhimoju R, Sherman W (2013). Protein and ligand preparation: parameters, protocols, and influence on virtual screening enrichments. J Comput Aided Mol Des.

[113] Jorgensen WL, Maxwell DS, Tirado-Rives J (1996). Development and testing of the OPLS all-atom force field on conformational energetics and properties of organic liquids. J Am Chem Soc.

[114] Halgren TA, Murphy RB, Friesner RA, Beard HS, Frye LL, Pollard WT, Banks JL (2004). Glide: A new approach for rapid, accurate docking and scoring. 2. Enrichment factors in database screening. J Med Chem.

[115] Farid R, Day T, Friesner RA, Pearlstein RA (2006). New insights about HERG blockade obtained from protein modeling, potential energy mapping, and docking studies. Bioorg Med Chem.

[116] Lyne PD, Lamb ML, Saeh JC (2006). Accurate prediction of the relative potencies of members of a series of kinase inhibitors using molecular docking and MM-GBSA scoring. J Med Chem.

